# Effect of p53 and its N‐terminally truncated isoform, Δ40p53, on breast cancer migration and invasion

**DOI:** 10.1002/1878-0261.13118

**Published:** 2021-11-23

**Authors:** Xiajie Zhang, Kira Groen, Brianna C. Morten, Luiza Steffens Reinhardt, Hamish G. Campbell, Antony W. Braithwaite, Jean‐Christophe Bourdon, Kelly A. Avery‐Kiejda

**Affiliations:** ^1^ Hunter Medical Research Institute New Lambton Heights NSW Australia; ^2^ School of Biomedical Sciences and Pharmacy College of Health, Medicine and Wellbeing The University of Newcastle NSW Australia; ^3^ Children’s Medical Research Institute University of Sydney NSW Australia; ^4^ Department of Pathology School of Medicine University of Otago Dunedin New Zealand; ^5^ Dundee Cancer Centre Ninewells Hospital and Medical School University of Dundee UK

**Keywords:** breast cancer, gene expression, migration and invasion, p53, Δ40p53

## Abstract

Breast cancer is the most diagnosed malignancy in women, with over half a million women dying from this disease each year. In our previous studies, ∆40p53, an N‐terminally truncated p53 isoform, was found to be upregulated in breast cancers, and a high ∆40p53 : p53α ratio was linked with worse disease‐free survival. Although p53α inhibits cancer migration and invasion, little is known about the role of ∆40p53 in regulating these metastasis‐related processes and its role in contributing to worse prognosis. The aim of this study was to assess the role of ∆40p53 in breast cancer migration and invasion. A relationship between Δ40p53 and gene expression profiles was identified in oestrogen‐receptor‐positive breast cancer specimens. To further evaluate the role of Δ40p53 in oestrogen‐receptor‐positive breast cancer, MCF‐7 and ZR75‐1 cell lines were transduced to knockdown p53α or Δ40p53 and overexpress Δ40p53. Proliferation, migration and invasion were assessed in the transduced sublines, and gene expression was assessed through RNA‐sequencing and validated by reverse‐transcription quantitative PCR. Knockdown of both p53α and ∆40p53 resulted in increased proliferation, whereas overexpression of ∆40p53 reduced proliferation rates. p53α knockdown was also associated with increased cell mobility. ∆40p53 overexpression reduced both migratory and invasive properties of the transduced cells. Phenotypic findings are supported by gene expression data, including differential expression of *LRG1*, *HYOU1*, *UBE2QL1*, *SERPINA5* and *PCDH7*. Taken together, these results suggest that, at the basal level, ∆40p53 works similarly to p53α in suppressing cellular mobility and proliferation, although the role of Δ40p53 may be cell context‐specific.

## Graphical abstract

P53 isoforms can affect the function of the p53 tetramer as a transcription factor. We investigated the effect of an altered Δ40p53 : p53 ratio on cell migration/invasion, as well as the transcriptome in breast cancer tissues and cell lines. ∆40p53‐overexpression reduced both migration and invasion suggesting that, at the basal level, ∆40p53 works similarly to p53α in suppressing cellular mobility.

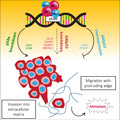

**Abbreviations**
B2M: β‐microglobulin
DBD: DNA‐binding domain
DEG: differentially expressed genes
ER+: oestrogen‐receptor‐positive
FDR: false discovery rate
FFPE: formalin‐fixed paraffin‐embedded
FGFR3: fibroblast growth factor receptor 3
GAPDH: glyceraldehyde 3‐phosphate dehydrogenase
GSEA: gene set enrichment analysis
HDM2: human double minute‐2
IDCs: invasive ductal carcinomas
IGF-1R: insulin growth factor 1 receptor
miRNA: microRNA
MMPs: matrix metalloproteinases
OD: oligomerisation domain
p53RE: p53 response element
RMA: robust multi‐array analysis
SD: standard deviation
shNT: nontargeting shRNA‐transduced cells serving as control
shRNA: short‐hairpin RNA
TAD: transactivation domain
wtp53α: wild‐type full‐length p53

## 1 Introduction

Breast cancer is the most commonly diagnosed malignancy in females and accounts for over half a million deaths in women worldwide [1]. *TP53* is the most frequently mutated gene in cancer. Wild‐type p53 suppresses tumorigenesis through multiple pathways, such as inducing cell‐cycle arrest, apoptosis, senescence and directly responding to oncogenic stress, whereas mutant p53 can gain dominant negative functions, contributing to tumorigenesis [2]. Intriguingly, p53 mutations are not ubiquitous in breast cancers (less than 25% of all cases) [3], indicating that other mechanisms are involved in ablating the canonical function of p53.

In 2005, it was discovered that p53 can be expressed as distinct protein isoforms other than the wild‐type full‐length p53 (wtp53α) [4]. Truncation can occur at the N terminus (Δ40p53, Δ133p53 and Δ160p53) or C terminus (α, β and γ) or both, giving rise to twelve isoforms retaining different proportions of the three main functional domains: transactivation domain (including TADI and II), DNA‐binding domain (DBD) and oligomerisation domain (OD) [4, 5, 6]. These domains are critical for p53α to function. The regulatory function of the truncated isoforms is not limited to their interaction with p53α [7]; Δ40p53α [8] and Δ133p53α [9] can function independently of p53α.

Δ40p53α (∆40p53 from here on) lacks the first 40 amino acids of p53α and can arise via two mechanisms. It has been reported by us and others that ∆40p53 is primarily generated by alternative splicing, which retains intron 2 encompassing several stop codons, thereby preventing p53α translation [10, 11]. Δ40p53 can also be produced by alternative translation at AUG40 [5]. The missing region encodes for TADI, resulting in immunity to human double minute‐2 (HDM2)‐mediated degradation and partial loss of transactivation ability [12]. The function of Δ40p53 is difficult to interpret, and different roles have been reported so far (for review, [13]).

Early studies showed that Δ40p53 heterotetramerisation led to elevated nuclear export of p53α, a reduction in the transactivation of certain p53α target genes and inhibition of p53α‐mediated apoptosis [5]. Hafsi *et al*. co‐transfected p53‐null cell lines with expression vectors for Δ40p53 and p53α at different amounts and measured the transactivation activity of a reporter gene containing the p53α response element of HDM2. They found that if Δ40p53 was expressed at levels higher than p53α (≥ 3 fold), Δ40p53 could inhibit p53α’s function, whereas lower or equivalent levels of Δ40p53 compared to p53α, had diverse effects that were cell line specific [12]. It is deducible that high levels of Δ40p53 compete with p53α in tetramer formation, and therefore, the canonical p53α function was compromised.

Other studies showed that overexpression of Δ40p53 induced apoptosis in melanoma cell lines [14] and reduced proliferation regardless of p53α status in hepatocellular carcinoma cell lines [15], where Δ40p53 stimulated the canonical function of p53α. Bourougaa *et al*. (2010) reported that Δ40p53 induced G2 arrest, while p53α induced G1 arrest, further establishing an independent role for Δ40p53 [16]. Using point mutations to inactivate phosphorylation residues, it was shown that TADI and TADII induce the expression of distinct genes, and hence, it is likely that the subset of genes transactivated by Δ40p53, which lacks TADI, is distinct from that of p53α [17]. Additionally, Δ40p53 has been shown to play a role in development. In mice, overexpression of Δ40p53 led to the activation of insulin growth factor 1 receptor (IGF‐1R), maintainence of proliferation and self‐renewal potential in embryonic stem cells during early development [18].

Δ40p53 is overexpressed in multiple cancers such as melanoma and breast cancer, and its expression has been correlated with prognostic and therapeutic outcomes [19, 20], suggesting a role for Δ40p53 in disease progression. Our group demonstrated the importance of Δ40p53 expression in breast cancer. It is the most highly expressed p53 isoform in breast cancer at the mRNA level, and it is significantly upregulated in tumours and cell lines compared to the normal breast. A high Δ40p53 : p53α ratio (> 0.7) is significantly associated with worse disease‐free survival (HR 2.713) [19]. These studies suggest that Δ40p53 may be involved in cellular functions that promote the aggressiveness of breast cancer, but functional studies demonstrating this are lacking, particularly on the endogenously expressed isoform.

As a tumour suppressor, p53α regulates the expression of a plethora of genes and microRNAs linked to the inhibition of proliferation, migration and invasion. For instance, p53α is known to regulate the expression of cell cycle regulatory genes *CDKN1A* and *RB1*, growth factor receptors *EGFR* and *MET*, and matrix metalloproteinases *MMP2* and *MMP9* [21], highlighting p53α’s regulatory role in proliferation and migration. P53α also induces the expression of miR‐34, which anatagonises *NOTCH*, *ZEB1* and *SNAI1*, further exemplifying how p53α promotes cell adhesion and reduces proliferation [22]. At the functional level, p53α’s role in repressing migration and invasion has also been shown [23]. Hence, there is a growing body of evidence suggesting a role for p53α in metastasis‐related processes; however, the role of ∆40p53 in this context has not been examined. In this study, we examined the role of Δ40p53 in migration, invasion and in the regulation of gene expression to determine whether this could provide an explanation for the association of high Δ40p53 : p53α with worse survival outcomes in breast cancer as identified in our previous study [19]. Our results showed that molecular inhibition of p53α was associated with increased cell mobility, confirming the previous association of loss of p53α function and increased metastatic potential. Overexpression of ∆40p53 reduced both migratory and invasive properties of the transduced cells. Inhibition of both p53α and ∆40p53 resulted in increased proliferation, while overexpression of ∆40p53 reduced proliferation rates. These phenotypic findings are supported by gene expression data. Taken together, these results suggest that at the basal level ∆40p53 works similarly to p53α in suppressing cellular mobility and proliferation.

## 2 Materials and methods

### 2.1 Breast cancer samples

Breast cancer samples were acquired from the Australian Breast Cancer Tissue Bank and have previously been described [19, 24]. All patients whose tissue samples were used in this study had an understanding of and provided written consent for the use of their tissue in research. mRNA that had been previously extracted from these samples was used in our studies. The study methodologies conformed to the standards set by the Declaration of Helsinki and were approved by the Hunter New England Human Research Ethics Committee (Approval number: 09/05/20/5.02).

### 2.2 RNA extraction

Total RNA was extracted from cells and formalin‐fixed paraffin‐embedded (FFPE) tissues using Trizol (Life Technologies, 15596026, Carlsbad, CA, USA).

### 2.3 Semiquantitative Real‐time RT‐PCR

cDNA was synthesised from 500 ng of total RNA using the High‐Capacity Reverse Transcription kit with RNase Inhibitor (Life Technologies, N8080119). Semiquantitative PCR was performed using Universal TaqMan Master Mix or Universal TaqMan Advanced Master Mix (RNA‐seq validation only) (Life Technologies, 4364340 and 4444965) on an ABI 7500 cycler or a Quant Studio 7 Pro PCR cycler (RNA‐seq validation only) with TaqMan gene expression assays (Life Technologies, 4331182): Δ40p53 [as previously described [19]] and *TP53* (Hs01034249_m1). Relative expression was determined using the 2^−ΔΔCt^ method [25] by normalising to the housekeeping gene β‐microglobulin (B2M) and glyceraldehyde 3‐phosphate dehydrogenase (GAPDH) as previously described [19]. Additionally, the expression of five differentially expressed genes identified by RNA‐seq (based on mean normalised counts and relevance) was confirmed by RT‐qPCR using the following probes: *LRG1* (Hs00364835_m1), *HYOU1* (Hs00197328_m1), *UBE2QL1* (Hs00331876_m1); *SERPINA5* (Hs04333915_m1) and *PCDH7* (Hs05574398_g1).

### 2.4 Human gene 1.0 array

100 ng of total RNA from FFPE samples was amplified (Ovation FFPE WTA kit) and biotinylated (Encore Biotin module) according to the manufacturers’ instructions (Nugen, San Carlos, CA, USA). The arrays were scanned on an Affymetrix GeneChip Scanner 3000 7G (Affymetrix, Santa Clara, CA, USA), the data were imported to Genomic Suite 6.6 (Partek, St. Louis, MO, USA), and a robust multi‐array analysis (RMA) was performed, which included log_2_ transformation, background correction, quantile normalisation and summarisation of the probe features resulting in a set of expression signal intensities. Unsupervised hierarchical clustering was performed on genes that were found to be differentially expressed in invasive ductal carcinomas (IDCs) with high ∆40p53 compared to IDCs with low ∆40p53 (*P* < 0.5; fold change > |1.5|). Correction for multiple testing was performed using the Benjamini–Hochberg procedure.

### 2.5 Cell culture

The human breast cancer cell lines MCF‐7 and ZR75‐1 (with wtp53α) were kind gifts from Professor Christine Clarke (Westmead Millennium Institute, University of Sydney, Australia) and Dr Judith Weidenhofer (The University of Newcastle, Australia), respectively. Before the beginning of the experiments, the cell lines were authenticated by the Australian Genome Research Facility (Fine Mapping and Custom Genotyping, 6173, 100% for MCF‐7 and 84.62% for ZR75‐1). Briefly, 1 × 10^6^ cells were collected and DNA was extracted by using Promega Genomic DNA purification Kit (Promega, A1120, Madison, WI, USA). GenePrint 10 system (Promega, B9510) was used to amplify 9 human loci from the extracted DNA and the amplified PCR products were used to generate a genetic profile, which was then compared to the profile provided by the supplier of the cell line. Cells were routinely cultured and passaged (up to the 20th passage after thawing) using DMEM [Life Technologies, 21063029), with 10% fetal bovine serum (FBS, Sigma‐Aldrich, F9423, St. Louis, MO, USA)], 200 mm l‐glutamine (Life Technologies, 25030081) and 2 µg·mL^−1^ insulin (Sigma‐Aldrich, 19278). Cells were kept in a cell culture incubator at 37 °C with 5% CO_2_ and humidity and routinely tested for mycoplasma according to the manufacture’s recommendations (MycoAlert PLUS, Lonza, LT07‐701).

### 2.6 shRNA transduction

Short‐hairpin RNAs (shRNAs) were designed to target Δ40p53α/β/γ isoforms (5’ AGACCTGTGGGAAGCGAAA 3’) or all other p53 isoforms (5’ GAAACTACTTCCTGAAAAC 3’) and cloned into pLKO.1‐puro‐CMV‐tGFP vectors (MISSION^®^ Lentiviral Transduction Particles, Sigma‐Aldrich) to generate specific and stable isoform knockdown in two oestrogen‐receptor‐positive breast cancer cell lines, MCF‐7 and ZR75‐1, expressing p53α. Our previous study has shown other than p53α and Δ40p53α, the mRNA expression levels of other p53 isoforms was extremely low (almost undetectable) in breast cancer cell lines [19, 20, 26], therefore, the shRNA‐mediated knockdown will primarily affect p53α and Δ40p53α expression, which will be referred to as shΔ40p53 and shp53α for simplicity. Cells were cultured with optimised concentrations of transducing particle units and 8 μg·mL^−1^ hexadimethrine bromide (Sigma‐Aldrich, H9268) for 24 to 48 h before changing to 1 μg·mL^−1^ puromycin‐supplemented media (puro‐media, puromycin from Sigma‐Aldrich, P9620) to select transduced cells. Cells were passaged in puro‐media for three more passages, until > 80% cells were GFP positive, prior to further validation. Transduced sublines were maintained in puro‐media. Using this method ∆40p53 knockdown sublines (‐sh∆40p53), p53α knockdown sublines (‐shp53α) and nontargeting control sublines (‐shNT) were established in both MCF‐7 and ZR75‐1 cell lines.

### 2.7 Vector transductions

Δ40p53‐overexpressing MCF‐7 cells were created by transducing MCF‐7 cells with a lentiviral expression construct containing Δ40p53. To generate the lentiviral construct, the empty vector LeGO‐iG2‐puro+ was linearised by digestion with BamH1 and Sbf1. Δ40p53 cDNA was amplified by PCR using primers, which incorporated 14 bps of sequence homologous to the LeGO‐iG2‐puro+ vector. The insert was then incorporated into the LeGO‐iG2‐puro+ vector by recombination (In‐Fusion, Clontech). The Δ40p53 lentivirus was then produced in 293T cells and used to transduce MCF‐7 cells. The resulting MCF‐7 cells were maintained in puro‐media as described above. Cells with stable Δ40p53‐overexpression are referred as MCF‐7‐Δ40p53 and MCF‐7‐LeGO (empty vector control).

### 2.8 Western blot

Western blot analysis was carried out as previously described [19, 20, 26]. A total of 30‐50 µg of protein extracts, obtained using 1% NP‐40 lysis buffer [50 mm Tris/HCl, 150 mm NaCl, 1% NP‐40, pH 8.0, 1× Mini Complete Protease Inhibitor EDTA‐free cocktail tablets (Roche Diagnostics, 11836153001)], were resolved on 8–12% SDS/PAGE gels and transferred onto nitrocellulose membranes. The membranes were blocked in Intercept^TM^ PBS Blocking Buffer (LI‐COR Biosciences, 927‐70001, Lincoln, NE, USA) and probed using primary antibody anti‐p53 (pantropic), clone DO‐1 (Merck Millipore, MABE327, Burlington, MA, USA) at a concentration of 1 µg·mL^−1^ (detects p53α, β and γ) and KJCA40 (provided by Dr Jean‐Christophe Bourdon) at a concentration of 2.5 µg·mL^−1^ (detects all Δ40p53 variants). GAPDH was used as a loading control (anti‐rabbit: Abcam, ab128915; anti‐mouse: Calbiochem, CB1001). Corresponding fluorescent secondary antibodies were purchased from LI‐COR Biosciences (Lincoln, 926‐32210 and 926‐68023). Blots were visualised and quantitated using the Odyssey CLx fluorescent imager (LI‐COR Biosciences, Lincoln, NE, USA) and Image Studio Lite v4.0 (LI‐COR Biosciences, Lincoln, NE, USA).

### 2.9 Proliferation assay

All MCF‐7 sublines were plated into 96‐well plates at a seeding density of 3000 cells·well^−1^ and monitored using the IncuCyte^TM^ ZOOM (Essen Bioscience, Ann Arbor, MI, USA). Confluence was calculated with integrated algorithms. All ZR75‐1 sublines were plated at a seeding density of 1 × 10^4^ cells·well^−1^ into four individual 96‐well plates to be processed every 24 h until 96 h. Cell Titer Glo^®^ 2.0 reagent (Promega, G9241) was applied to each plate according to the manufacturer’s instructions and luminescence was measured using a Cytation 3 (BioTek, Winooski, VT, USA). All experiments were repeated three times on separate days with three technical replicates.

### 2.10 Migration/Invasion assay based on the wound healing method

Migration and invasion were measured with the wound healing assay as previously described [27]. Briefly, a coated (100 μL·mL^−1^ matrigel, Sigma‐Aldrich, E6909) 96‐well plate (invasion assay) or an uncoated 96‐well plate (migration assay) was seeded with cells overnight to achieve a confluent cell monolayer for the next day prior to the scratch. Plates for the migration/invasion assay were scratched using the 96‐well WoundMaker^TM^ (Essen Bioscience). Dislodged cell sheets were washed away with prewarmed media and fresh media was added (migration assay). For the invasion assay, matrigel was diluted to 125 μg·mL^−1^ with cold fresh media as previously described [28] and 50 μL was added to wells before gelation. Wounded cells were placed into the IncuCyte (Essen Bioscience) and monitored until the wounds closed.

### 2.11 Transwell migration assay

All cell lines were cultured to 80% confluence and resuspended in DMEM. 1 × 10^5^ cells were seeded into transwell inserts (polycarbonate membrane with 8‐μm pores, 24‐well format, Sigma‐Aldrich, CLS3422) in triplicate, and allowed to migrate into the lower chamber with puro‐media (10% FBS) for 24 h (MCF‐7 sublines) or 48 h (ZR75‐1 sublines) under standard cell culture conditions. Cells were fixed with 3.7% formaldehyde at room temperature for 15 min and cells on the upper side of the transwell inserts were removed before the inserts were stained with crystal violet. Migrated cells were counted under a light microscope with a 10x objective. Cell numbers in five random fields of view were averaged per insert. All experiments were repeated three times on separate days.

### 2.12 Transwell invasion assay

Transwell inserts were coated with 125 μg·mL^−1^ matrigel overnight under standard cell culture conditions. Transduced MCF‐7 and ZR75‐1 sublines were cultured to 80% confluence and resuspended in DMEM. 1 x 10^5^ cells were seeded into transwell inserts in triplicate and allowed to migrate into the lower chamber with puro‐media (10% FBS) for 24 h (MCF‐7 sublines) or 48 h (ZR75‐1 sublines) under standard cell culture conditions. Transwell inserts and invaded cell numbers were processed as described in the migration assay. All experiments were repeated three times on separate days.

### 2.13 RNA‐seq

All sublines were seeded in triplicate into 6‐well plates for 24 h (3–4 × 10^5^ to achieve around 70% confluence the next day). RNA was extracted from the harvested cells using Trizol. RNA libraries were prepared using the Illumina TruSeq stranded mRNA library prep kit (Illumina, 20020594, San Diego, CA, USA). Pooled libraries were loaded onto a NextSeq 500/550 High Output Flow Cell (single‐end, 75 cycles, Illumina, FC‐404‐2005) and run on a NextSeq 500 system (Illumina). FASTQ files were generated by BaseSpace (Illumina) and mapped to Human GRCh37 Assembly using STAR [29]. Differential expression was computed in DESeq2. Genes of ≥ 50 counts, log_2_(fold change) ≥ |1|, and a false discovery rate (FDR)‐adjusted *P*‐value ≤ 0.05 were deemed to be differentially expressed.

### 2.14 Gene set enrichment analysis

Gene set enrichment analysis (GSEA) was performed by Enrichr [30] using GO Biological Process 2018.

### 2.15 Statistical analyses

For cell line experiments, unpaired student t‐tests were performed for two comparisons, and one‐way ANOVA or two‐way ANOVA for multiple comparisons, corrected for multiple comparisons using the Dunnett’s or Sidak’s test, respectively. All results are the mean of three independent experiments, and error bars represent the standard deviation (SD) or are otherwise indicated. All statistical analyses were performed using graphpad Prism v. 6.0. An adjusted *P*‐value of < 0.05 was considered statistically significant.

## 3 Results

### 3.1 Differential gene expression in ER+ IDCs with high vs low ∆40p53

**Fig. 1:**
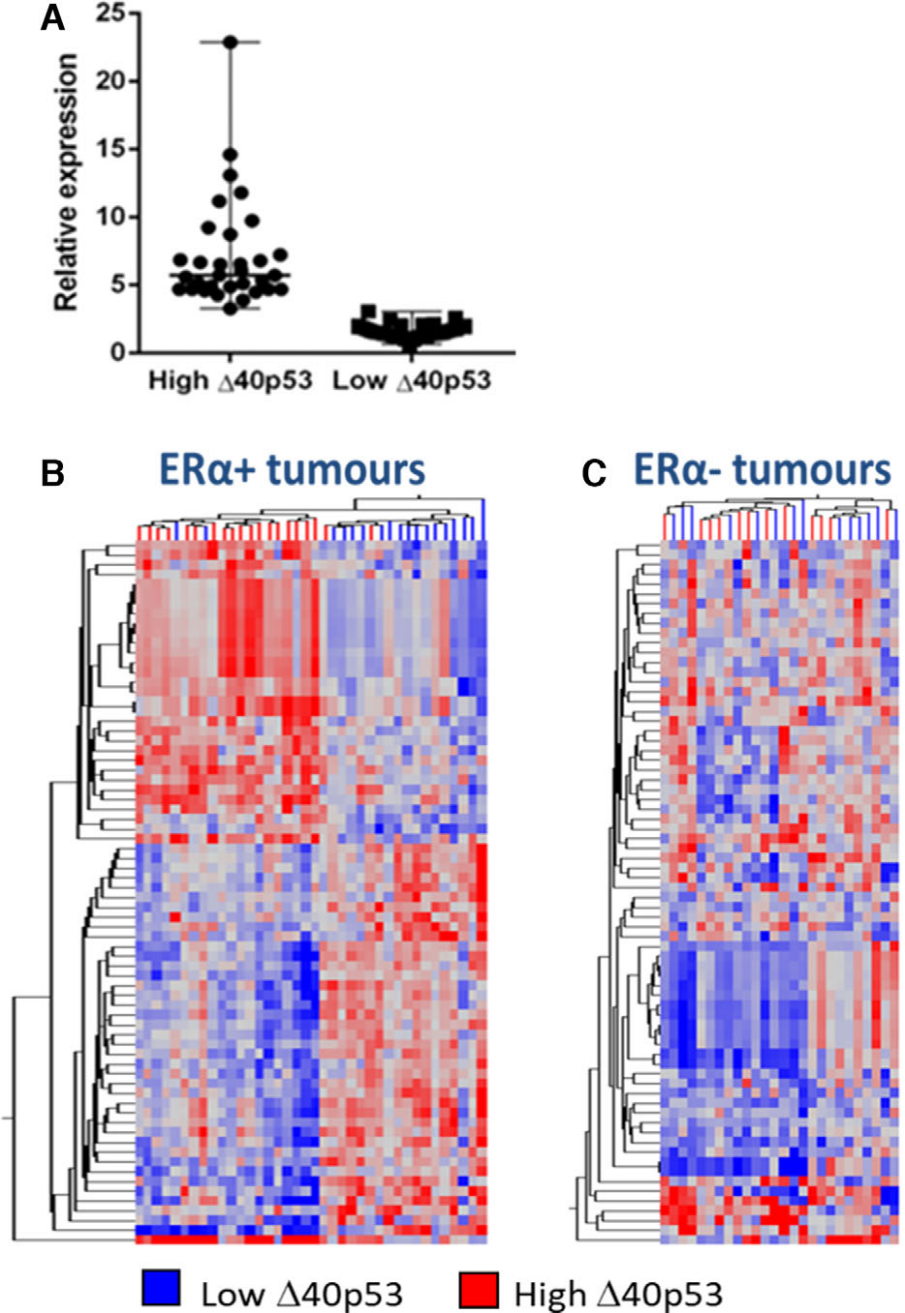
High Δ40p53 expression is associated with altered gene expression in 38 ERα+ but not 16 ER‐ breast tumours. (A) 38 ER+ and 16 ER‐ breast tumours were divided into high and low Δ40p53 expression as determined by RT‐qPCR using median ∆40p53 expression as the cut‐off. Experiments were performed in three technical replicates. (B) The expression of 28 869 genes was analysed by gene expression array in the tumour samples. Hierarchical clustering was performed on 72 transcripts found to be differentially expressed in high (red branches) vs low (blue branches) ∆40p53‐expressing ERα+ tumours. (C) The 72 differentially expressed transcripts were hierarchically clustered in ER‐ breast cancers in high (red) vs low (blue) ∆40p53‐expressing tumours. Similarity in the expression between genes (branches on left) and between samples (branches on top) was measured using Euclidean correlation. Distances between clustered branches represent the average distance. Upregulated expression is represented by red, downregulated expression is represented by blue, and equal expression is represented by grey.

Using RT‐qPCR, Δ40p53 expression levels were measured in 38 oestrogen‐receptor‐positive (ER+) and 16 ER‐ IDCs (Grade 1 and 2) for which gene expression data has been previously published by our group [24]. To determine genes that are affected by endogenous ∆40p53, both ER+ and ER‐ IDCs were classified based on high or low ∆40p53 expression (as compared to the median expression level of all IDC cases) (Fig. 1A). All breast cancer samples were then subjected to HumanGene1.0 Array (Affymetrix) analysis. In ER+ breast cancer samples, 72 transcripts (59 annotated genes, Table 1) were differentially expressed in tumours expressing high ∆40p53 vs low ∆40p53 (> |1.5|‐fold, *P < *0.05, FDR 5%) (Fig. 1B). The same pattern of differential expression was not observed in ER‐ breast cancer cases (Fig. 1C). Thus, the transcriptional effects of ∆40p53 may be ER‐dependent. GSEA revealed GO terms associated with immune responses mediated by cytokines, which is not surprising given the fact that the breast cancer specimens contain all cell types including the tumour cells, stroma, epithelium and the lymphatic cells. Additionally, genes involved in extracellular matrix organisation, such as *ACTN1* (actinin 1), *FBLN1* (fibulin 1) and *ITGB2* (integrin 2), were highlighted by GSEA, indicating endogenously higher levels of Δ40p53 are associated with downregulated cell mobility (Table 2).

**Table 1:** 59 annotated differentially expressed genes in ER+ breast cancers.

Gene	Gene description	Regulation by high Δ40p53	FC (abs)	*P‐*value
C14orf174	Chromosome 14 open reading frame 174	Up	1.632	0.006
C5orf30	Chromosome 5 open reading frame 30	Up	1.580	0.045
CCDC125	Coiled‐coil domain containing 125	Up	1.636	0.014
CYFIP2	Cytoplasmic FMR1 interacting protein 2	Up	1.673	0.043
DNAJC12	DnaJ (Hsp40) homolog, subfamily C, member 12	Up	2.340	0.029
EFHC1	EF‐hand domain (C‐terminal) containing 1	Up	1.528	0.015
FAM174A	Family with sequence similarity 174, member A	Up	1.515	0.021
GUSBP1	Glucuronidase, beta pseudogene 1	Up	1.609	0.009
GUSBP3	Glucuronidase, beta pseudogene 3	Up	1.773	0.008
HSD17B7	Hydroxysteroid (17‐beta) dehydrogenase 7	Up	1.720	0.011
HSD17B7P2	Hydroxysteroid (17‐beta) dehydrogenase 7 pseudogene 2	Up	1.719	0.045
KIF3A	Kinesin family member 3A	Up	1.511	0.010
KLHDC1	Kelch domain containing 1	Up	1.518	0.030
MCCC2	Methylcrotonoyl‐CoA carboxylase 2 (beta)	Up	1.618	0.036
MIPOL1	Mirror‐image polydactyly 1	Up	1.632	0.021
NUCB2	Nucleobindin 2	Up	1.541	0.021
NUDT12	Nudix (nucleoside diphosphate linked moiety X)‐type motif 12	Up	1.592	0.027
SNORA48	Small nucleolar RNA, H/ACA box 48	Up	1.533	0.012
SSBP2	Single‐stranded DNA binding protein 2	Up	1.578	0.027
ACTN1	Actinin, alpha 1	Down	1.515	0.016
ARL4C	ADP‐ribosylation factor‐like 4C	Down	1.654	0.005
C1R	Complement component 1, or subcomponent	Down	1.778	0.022
CCR1	Chemokine (C‐C motif) receptor 1	Down	1.586	0.041
CD4	Cluster of differentiation 4	Down	1.513	0.034
CERCAM	Cerebral endothelial cell adhesion molecule	Down	1.525	0.018
CHI3L1	Chitinase 3‐like 1 (cartilage glycoprotein‐39)	Down	2.532	0.013
CHST11	Carbohydrate (chondroitin 4) sulfotransferase 11	Down	1.517	0.019
CNN2	Calponin 2	Down	1.708	0.013
CTSD	Cathepsin D	Down	1.760	0.039
CYB5R3	Cytochrome b5 reductase 3	Down	1.533	0.004
DPP4	Dipeptidyl‐peptidase 4	Down	2.048	0.039
FBLN1	Fibulin 1	Down	1.648	0.043
FLNA	Filamin A, alpha	Down	1.688	0.004
FPR1	Formyl peptide receptor 1	Down	1.513	0.006
GREM1	Gremlin 1, cysteine knot superfamily, homolog (Xenopus laevis)	Down	1.847	0.027
HAS2	Hyaluronan synthase 2	Down	1.763	0.030
ITGB2	Integrin, beta 2 (complement component 3 receptor 3 and 4 subunit)	Down	1.564	0.037
KCNJ15	Potassium inwardly‐rectifying channel, subfamily J, member 15	Down	1.547	0.032
LAPTM5	Lysosomal protein transmembrane 5	Down	1.612	0.013
LGALS1	Lectin, galactoside‐binding, soluble, 1	Down	1.630	0.013
LILRB4	Leukocyte immunoglobulin‐like receptor, subfamily B (with TM and ITIM domains), member 4	Down	1.572	0.016
LRP1	Low‐density lipoprotein receptor‐related protein 1	Down	1.790	0.014
MFGE8	Milk fat globule‐EGF factor 8 protein	Down	1.732	0.007
MMP9	Matrix metallopeptidase 9 (gelatinase B, 92 kDa gelatinase, 92 kDa type IV collagenase)	Down	1.680	0.038
MYL9	Myosin, light chain 9, regulatory	Down	1.539	0.032
PCOLCE	Procollagen C‐endopeptidase enhancer	Down	1.778	0.030
PFKFB3	6‐phosphofructo‐2‐kinase/fructose‐2,6‐biphosphatase 3	Down	1.597	0.032
PLAUR	Plasminogen activator, urokinase receptor	Down	1.554	0.015
PLTP	Phospholipid transfer protein	Down	1.616	0.027
SERPING1	Serpin peptidase inhibitor, clade G (C1 inhibitor), member 1	Down	1.504	0.045
SIRPB1	Signal‐regulatory protein beta 1	Down	1.537	0.047
SLC43A3	Solute carrier family 43, member 3	Down	1.579	0.010
SRPX	Sushi‐repeat‐containing protein, X‐linked	Down	1.817	0.027
STARD3	StAR‐related lipid transfer (START) domain containing 3	Down	1.645	0.030
TAGLN	Transgelin	Down	1.697	0.032
TGM2	Transglutaminase 2 (C polypeptide, protein‐glutamine‐gamma‐glutamyltransferase)	Down	1.630	0.008
TIMP1	TIMP metallopeptidase inhibitor 1	Down	1.572	0.013
TMEM45A	Transmembrane protein 45A	Down	1.867	0.047
TNFRSF21	Tumour necrosis factor receptor superfamily, member 21	Down	1.744	0.004

**Table 2:** Gene set enrichment analysis of 59 annotated DEGs in ER+ breast cancers.

GO biological Process Term	*P*‐value	Adjusted *P*‐value	Odds Ratio	Combined Score	Genes
neutrophil mediated immunity (GO : 0002446)	1.21E‐05	0.01	6.25	70.78	CNN2;CYB5R3;ITGB2;PLAUR;FPR1;CHI3L1;CTSD;MMP9;SIRPB1
neutrophil activation involved in immune response (GO : 0002283)	1.13E‐05	0.01	6.30	71.78	CNN2;CYB5R3;ITGB2;PLAUR;FPR1;CHI3L1;CTSD;MMP9;SIRPB1
negative regulation of cellular component movement (GO : 0051271)	1.08E‐05	0.02	67.80	775.20	ACTN1;CCDC125;FBLN1
neutrophil degranulation (GO : 0043312)	1.06E‐05	0.03	6.36	72.80	CNN2;CYB5R3;ITGB2;PLAUR;FPR1;CHI3L1;CTSD;MMP9;SIRPB1
cellular response to cytokine stimulus (GO : 0071345)	7.16E‐06	0.04	6.68	79.09	CCR1;CD4;ITGB2;FPR1;CHI3L1;HAS2;TIMP1;MMP9;TNFRSF21
positive regulation of viral entry into host cell (GO : 0046598)	2.37E‐04	0.20	84.75	707.47	CD4;LGALS1
regulation of cysteine‐type endopeptidase activity involved in apoptotic signalling pathway (GO : 2001267)	3.04E‐04	0.22	75.33	610.08	PLAUR;MMP9
cytokine‐mediated signalling pathway (GO : 0019221)	5.20E‐04	0.24	4.28	32.35	CNN2;CCR1;CD4;ITGB2;FPR1;TIMP1;MMP9;TNFRSF21
extracellular matrix organisation (GO : 0030198)	5.82E‐04	0.25	7.37	54.90	GREM1;ITGB2;HAS2;TIMP1;MMP9
positive regulation of cholesterol efflux (GO : 0010875)	6.54E‐04	0.26	52.15	382.43	LRP1;PLTP

Blue genes are downregulated, and red genes are up regulated in breast cancer specimens with high ∆40p53 compared to low ∆40p53.

### 3.2 Establishment of ∆40p53 and p53α knockdown in the ER+ cell lines MCF‐7 and ZR75‐1

**Fig. 2:**
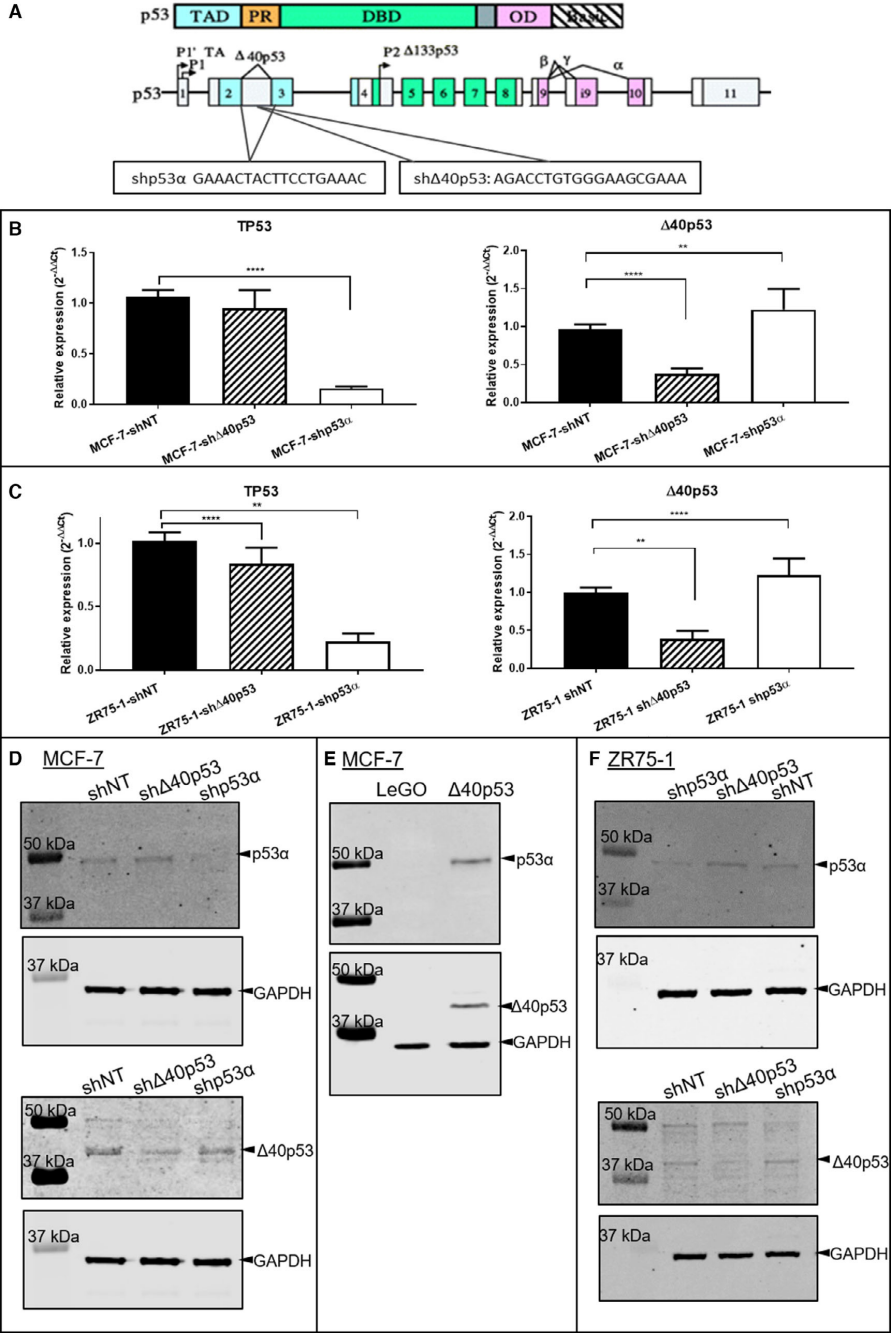
Overexpression and knockdown of Δ40p53 in breast cancer cell lines. (A) The p53 protein includes a transactivation domain (TAD, blue), a DNA‐binding domain (DBD, green) and an oligomerisation domain (OD, purple). The p53 gene has 11 exons. Δ40p53 lacks part of the TAD but includes part of intron 2, which shΔ40p53 targets. Shp53α targets the sequence that spans across exon 2/3, therefore generating isoform‐specific knockdown. Knockdown of p53α and Δ40p53 was quantitated at the mRNA level in MCF‐7 (B) and ZR75‐1 (C) derived cell lines. mRNA expression levels were measured using semiquantitative real‐time PCR. All real‐time PCR results were normalised to the housekeeping gene *GAPDH*, and transduction conditions were compared to the nontargeting shRNA control (shNT). Relative expression was calculated using 2^−ΔΔCt^ method as described [25]. Experiments were repeated three times in three technical replicates. Results are the mean of three independent experiments, and error bars represent the standard deviation (SD). Significant differences are indicated with brackets and stars by one‐way ANOVA. **P < 0.01, ****P < 0.0001. p53α and Δ40p53 protein levels were detected by Western blot in three independent experiments using DO‐1 (detecting p53α) and KJCA40 (detecting Δ40p53) antibodies, respectively. The protein expression levels of p53α and Δ40p53 are shown by representative Western blots in the MCF‐7 sublines (D) including the pre‐established Δ40p53‐overexpression cells (MCF‐7‐Δ40p53 and its control MCF‐7‐LeGO (E) and the shRNA‐transduced sublines, as well as the transduced ZR75‐1 sublines (F).

To further investigate the role of ∆40p53 in ER+ breast cancer, knockdown sublines were established through shRNA transduction. ER+ MCF‐7 and ZR75‐1 cell lines were transduced with shRNA vectors against p53α (‐shp53α), ∆40p53 (‐sh∆40p53) and a nontargeting control (‐shNT) (Fig. 2A). The region targeted by the p53 shRNA (exon 2/3 junction) will inhibit the expression of all p53 isoforms with the exception of ∆40p53 (and other N‐terminal variants, i.e. ∆133p53 and ∆160p53, that are transcribed from the P2 promoter). The region targeted by the ∆40p53 shRNA (intron 2) will result in the inhibition of all ∆40p53 transcripts (regardless of whether their C terminus is full‐length (α) or truncated (β/γ)) that are generated by alternative splicing. Additionally, a ∆40p53 overexpression model was also established by transfecting MCF‐7 cells with a lentiviral construct containing the open reading frame of ∆40p53 (MCF‐7‐∆40p53), or an empty lentiviral construct (MCF‐7‐LeGO), which served as a control. The Δ40p53 cDNA lacks introns; hence, only the full‐length Δ40p53α will be overexpressed in the MCF‐7 cell line.

shp53α was able to inhibit the mRNA expression of p53α, but not Δ40p53 in both MCF‐7 and ZR75‐1 cells by approximately 80 and 75%, respectively (Fig. 2B,C). This reduced expression was confirmed at the protein level with the DO‐1 antibody (Fig. 2D,F). Transduction with shΔ40p53 did not change the mRNA expression level of p53α but knocked down Δ40p53 specifically by 65% in MCF‐7 cells and 55% in ZR75‐1 cells (Fig. 2B,C). These results were confirmed at the protein level with the KJCA40 antibody (Fig. 2D,F). Interestingly, in the MCF‐7‐shp53α subline, the Δ40p53 mRNA level was increased by 1.4‐fold compared to the nontargeting control (Fig. 2B).

In the overexpression model (MCF‐7‐∆40p53), increased levels of p53α and ∆40p53 were detected (Fig. 2E), consistent with the stabilising effects of Δ40p53 on p53α [14].

### 3.3 ∆40p53 knockdown alters the morphology of ZR75‐1 cells

**Fig. 3:**
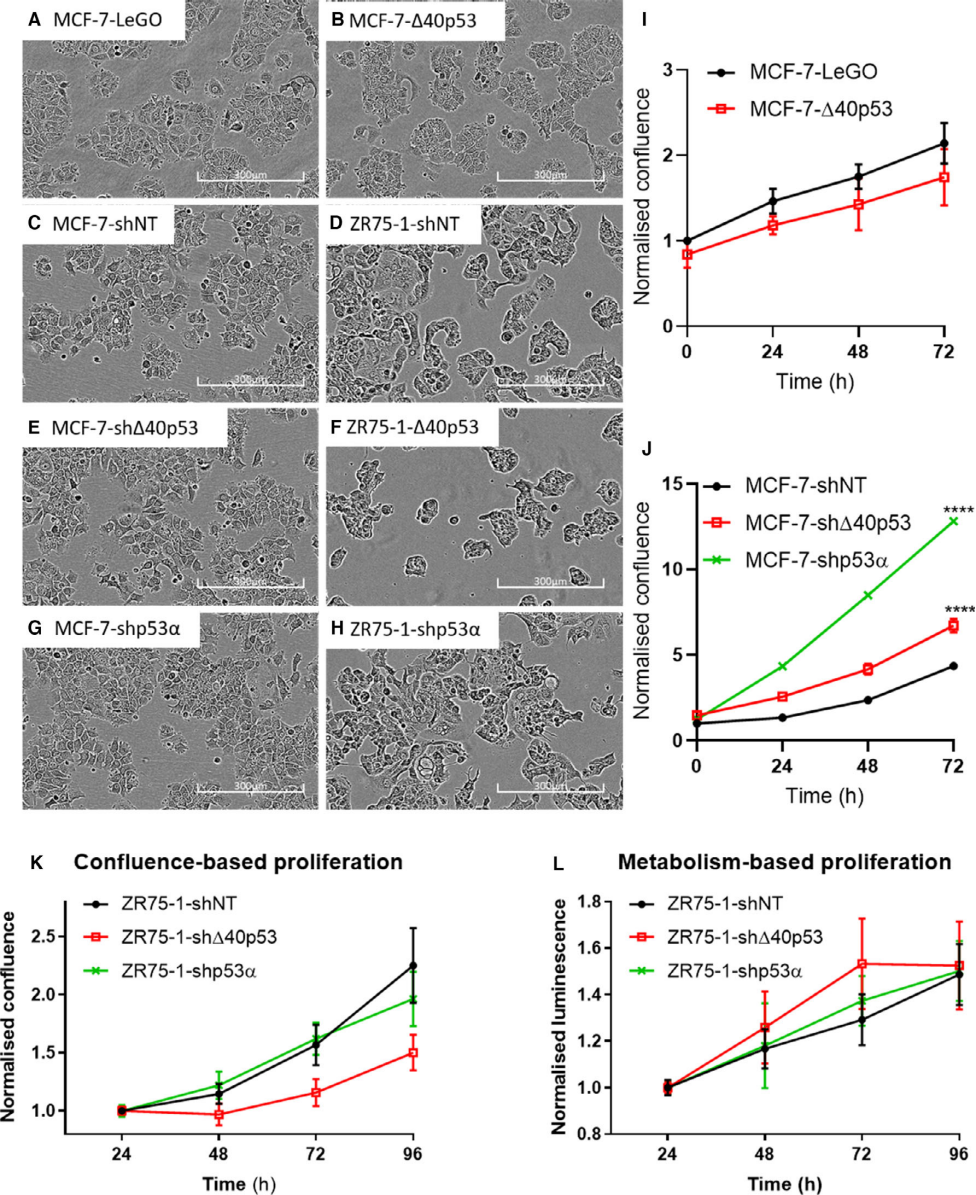
Δ40p53 can alter cell morphology in ZR75‐1 sublines and altered Δ40p53 and p53α can alter cell proliferation in MCF‐7 sublines. The top panel shows the high‐resolution image of Δ40p53‐overexpressing MCF‐7 cells (MCF‐7‐Δ40p53) (B) and their empty vector control (MCF‐7‐LeGO) (A, C, E and G) show stable knockdown of Δ40p53 and p53α in MCF‐7 cells as well as the nontargeting control. (D, F and H) show stable knockdown of Δ40p53 and p53α in ZR75‐1 cells as well as the nontargeting control. Images were taken by IncuCyte equipped with a 10x objective. Representative images of the sublines parental cells are shown in Fig. S1. Cell proliferation was measured by confluence using the IncuCyte in Δ40p53‐overexpressing MCF‐7 sublines (I) and Δ40p53/p53α knockdown MCF‐7 sublines (J). Cell proliferation of ZR75‐1 sublines was measured by confluence using the IncuCyte (K) and by metabolism using CellTiter Glo^®^ (L), normalising to the value of 24 h within each subline. Results are the mean of three independent experiments in triplicate and error bars indicate the standard deviation (SD). Unpaired t‐tests and one‐way ANOVA were used to identify significance. ****P < 0.0001.

Following successful knockdown of Δ40p53 or p53α by shRNA transduction in MCF‐7 and ZR75‐1 cells and overexpression of ∆40p53 in MCF‐7 cells, we determined if the long‐term knockdown/overexpression resulted in overt morphological changes. All MCF‐7 sublines displayed identical morphology to the parental MCF‐7 cells (Fig. S1A) and were able to form confluent monolayers despite altered Δ40p53/p53α expression (Fig. 3A,B,C,E,G). In contrast, ZR75‐1 sublines (Fig. 3D,F,H) showed distinct morphological differences. ZR75‐1‐shNT and ZR75‐1‐shp53α cells (Fig. 3D,H) had identical morphology to the parental ZR75‐1 cells (Fig. S1B), but ZR75‐1‐shΔ40p53 cells (Fig. 3F) aggregated into islands of cells and lost the ability to form a monolayer.

### 3.4 ∆40p53 affects proliferation in a cell context‐specific manner

Proliferation was firstly evaluated in all sublines to determine the role of Δ40p53 and p53α in cell growth. In MCF‐7 cells, Δ40p53‐overexpression led to an indicative but not significant reduction in proliferation compared to the control subline (Fig. 3I), while Δ40p53 knockdown led to significant increased proliferation (Fig. 3J). Similarly, knockdown of p53α in MCF‐7 cells led to significant increased proliferation, consistent with its role as a tumour suppressor [2]. In comparison, similar growth rates were observed between ZR75‐1‐shNT and ZR75‐1‐shp53α sublines, but the ZR75‐1‐shΔ40p53 subline had a significantly slower growth rate when compared by the confluence‐based assay (Fig. 3K). However, confluence‐based proliferation assays rely on the ability of cells of identical morphology to form a confluent monolayer. The unique morphologies amongst the ZR75‐1 sublines, especially the shΔ40p53 subline, may therefore have affected confluence‐based proliferation assays. Therefore, a metabolic proliferation assay (Cell Titer Glo^®^ 2.0 end‐point assay) was used to measure proliferation in ZR75‐1 sublines. The metabolic proliferation assay indicated that there was no difference in proliferation between ZR75‐1 sublines (Fig. 3L). The above results showed that Δ40p53 had cell context‐specific effects on proliferation, decreasing proliferation in MCF‐7 cells, but not ZR75‐1 cells, even though both cell lines contain wtp53 and are ER+.

### 3.5 P53α knockdown enhances cell mobility, while ∆40p53 overexpression impairs it

**Fig. 4:**
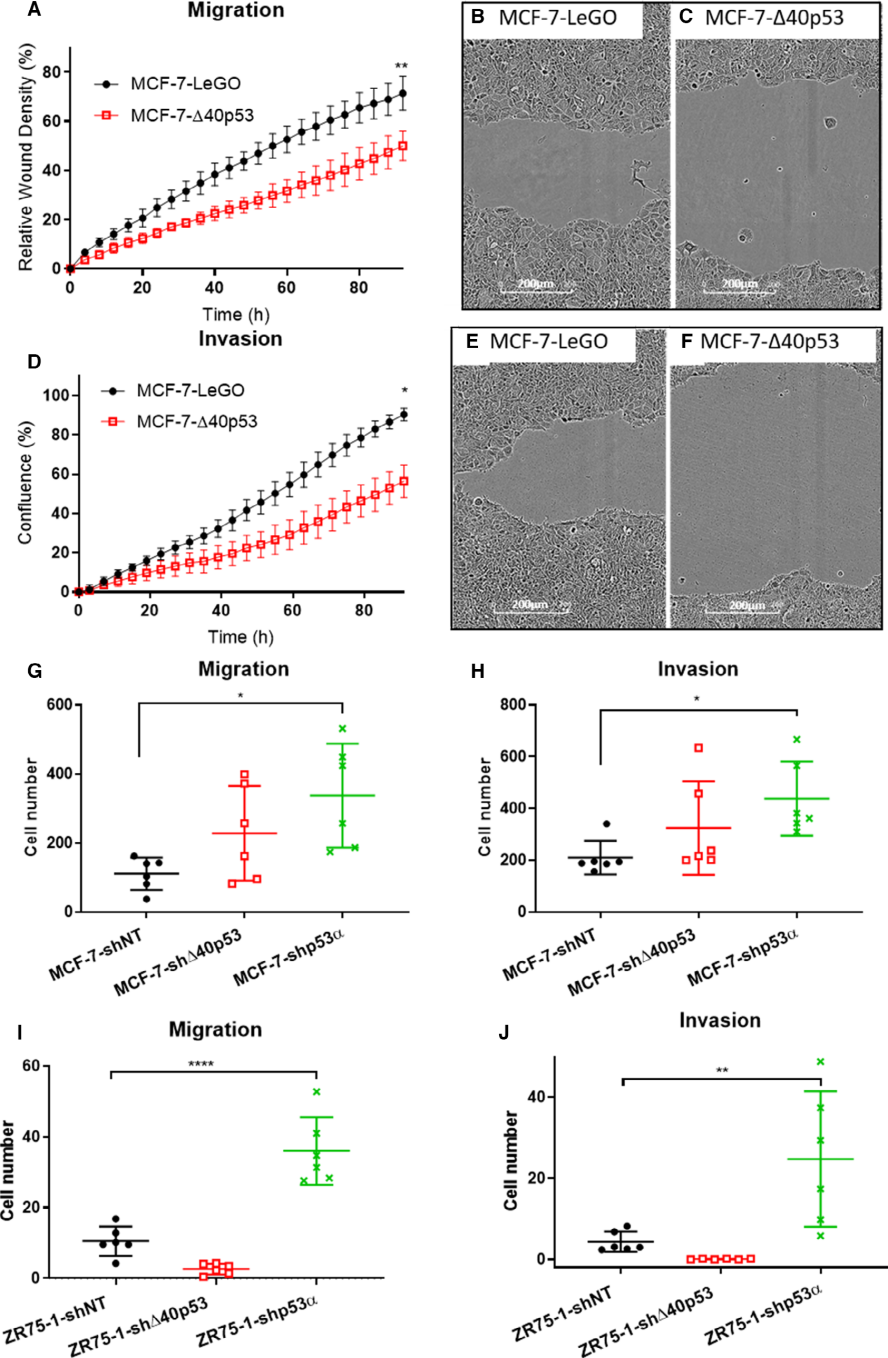
Δ40p53 and p53α can alter cell migration and invasion in MCF‐7 and ZR75‐1 cells. The metric Relative Wound Density was used to quantitate cell migration (A) and invasion (D). (B and C) at 72 h after scratch wounds were made in the migration assay. The wound width of MCF‐7‐Δ40p53 cells was larger than that of MCF‐7‐LeGO cells and the migratory front of MCF‐7‐Δ40p53 cells appeared less active than that of MCF‐7‐LeGO cells. (E and F) at 72 h after wounds had been made in the invasion assay, MCF‐7‐Δ40p53 cells showed impaired invasion (F) compared to MCF‐7‐LeGO cells (E). Experiments were repeated three times in triplicate. Representative results and images are shown. In MCF‐7 cells, transwell migration (G) and invasion (H) showed no significant increase in cell mobility when Δ40p53 was knocked down but increased cell mobility when p53α was knocked down. In ZR75‐1 cells, transwell migration (I) and invasion (J) showed no significant increase in cell mobility when Δ40p53 was knocked down but increased cell mobility when p53α was knocked down. Results are the mean of three independent experiments, and error bars represent the standard deviation of the mean (SD). Experiments were repeated three times in triplicate. Significant differences are indicated with brackets and stars by one‐way ANOVA. *P < 0.05, **P < 0.01, ****P < 0.0001.

Cell mobility was assessed through the scratch wound assay and the Transwell assay, both of which can be modified to analyse migration (no matrigel coating) or invasion (with matrigel coating). MCF‐7 cells overexpressing Δ40p53 migrated slower compared to MCF‐7‐LeGO cells (empty control vector, Fig. 4A–C). Both sublines exhibited enlarged cells at the migratory front and protruding edges stretching towards the cell‐free area, but the wound remained significantly larger in MCF‐7‐∆40p53 cells (Fig. 4B,C). Moreover, MCF‐7‐Δ40p53 cells had greatly impaired invasion capacity and phenotype compared to MCF‐7‐LeGO cells (Fig. 4D–F).

We then sought to assess cell migration/invasion in the shRNA‐transduced MCF‐7 sublines. However, shRNA knockdown of ∆40p53 and p53α altered the cells’ ability to form a uniform confluent monolayer overnight (which was achievable in MCF‐7‐LeGO and MCF‐7‐∆40p53, Fig. 3A,B). Hence, achieving similar confluence required for wound healing assays was challenging. To circumvent this issue, transwell assays were used, which are based on equal seeding density and do not require similar confluence to be established. The number of cells that migrated (Fig. 4G) or invaded (Fig. 4H) through the membrane of the transwell inserts was not significantly different between MCF‐7‐shΔ40p53 cells and MCF‐7‐shNT cells. In contrast, MCF‐7‐shp53α cells had acquired significantly increased cell mobility, indicating p53α as the major modulator of migration and invasion in MCF‐7 cells (Fig. 4G,H).

ZR75‐1 cells had 10‐fold less mobility than MCF‐7 cells in migration and invasion assays (Fig. 4G–J). However, consistent with the findings in MCF‐7 cells, ZR75‐1‐shp53α cells had significantly increased migratory and invasive capability compared to ZR75‐1‐shNT cells. ZR75‐1‐shΔ40p53 cells had the least cell mobility, albeit not significantly changed relative to the shNT control cells (Fig. 4I,J).

Thus, knockdown of p53α resulted in significantly increased cell migration and invasion in both MCF‐7 and ZR75‐1 cells, demonstrating a dominant role of the full‐length isoform in these processes. In contrast, Δ40p53‐overexpression led to reduced cell mobility, while Δ40p53 knockdown had no significant effect on mobility in MCF‐7 or ZR75‐1 cell lines.

### 3.6 Molecular profiles support a proliferative, migratory and invasive phenotype

**Fig. 5:**
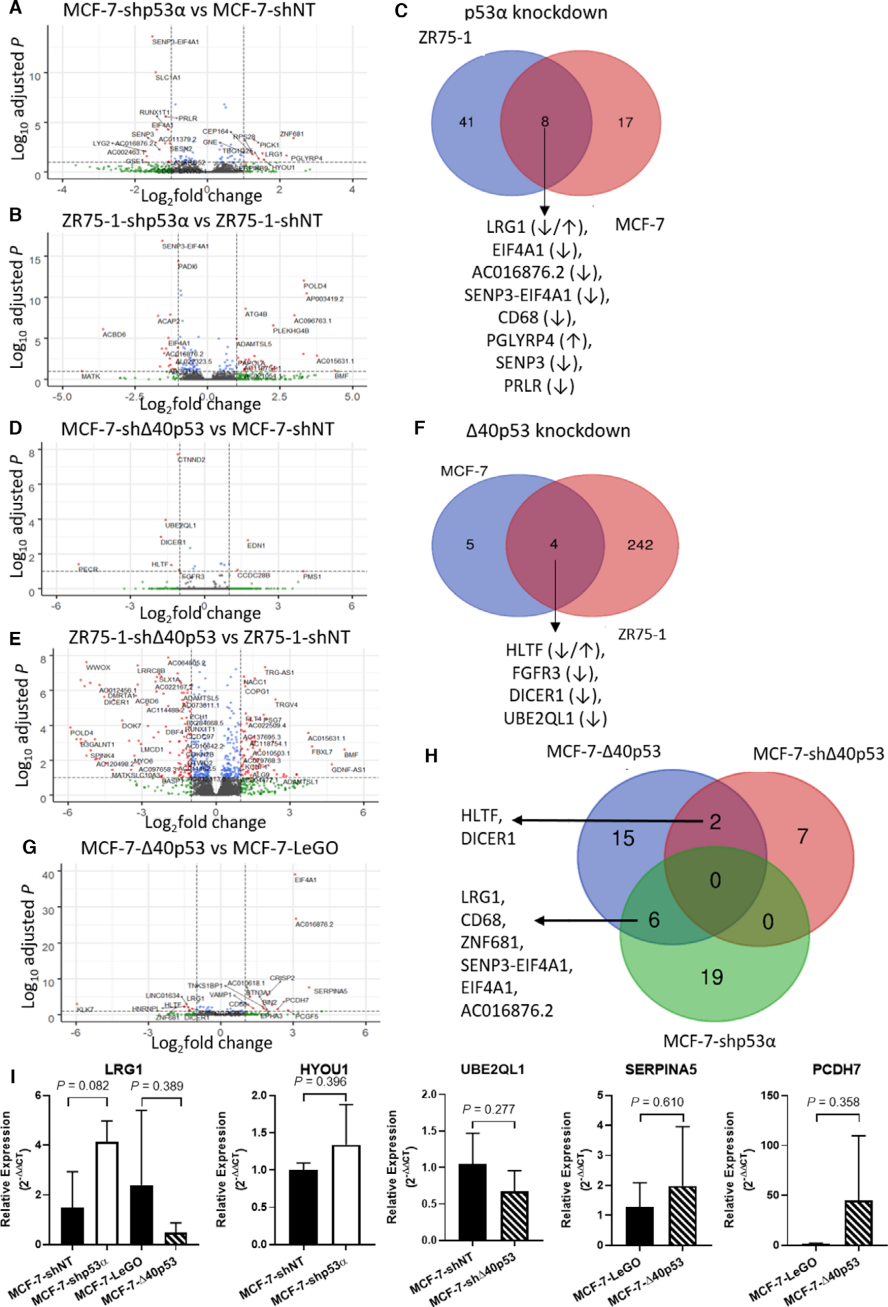
Differential gene expression in Δ40p53 and p53α knockdown sublines and the Δ40p53 overexpression subline. Differential gene expression in the different isoform knockdown and overexpression sublines relative to their respective control sublines are highlighted through Volcano plots in which dotted lines represent an false discovery rate (FDR)‐adjusted *P*‐value cut‐off of 0.05 and a log_2_(fold change) cut‐off of |1|. Differentially expressed genes are highlighted in red for MCF‐7‐shp53α vs MCF‐7‐shNT (A), ZR75‐1‐shp53α vs ZR75‐1‐shNT (B), MCF‐7‐∆40p53 vs MCF‐7‐shNT (D), ZR75‐1‐sh∆40p53 vs ZR75‐1‐shNT (E), and MCF‐7‐∆40p53 vs MCF‐7‐LeGO (G). Overlap between DEGs in the different sublines is minimal (C, F, H), with arrows indicating up/down regulation of the respective genes. Differential expression of a subset of genes (based on normalised gene counts and relevance) was validated by RT‐qPCR (I). Results are the mean of three independent experiments in triplicate and error bars indicate SD. Unpaired t‐tests were used to identify significance and *P*‐values are shown above the brackets.

To evaluate the molecular mechanisms driving differences in proliferation, migration and invasion between Δ40p53 knockdown/overexpression, p53α knockdown and control sublines, RNA was extracted from each subline and subjected to RNA‐seq. In both MCF‐7 and ZR75‐1‐derived sublines, knockdown of p53α altered the expression of less than 1% of the genes detected by RNA‐seq, and the overlap of differentially expressed genes (DEGs) between the two sublines was limited to eight genes (Fig. 5A–C, Table S1). Of the shared DEGs between the p53α‐knockdown sublines, five of the downregulated genes (*EIF4A1*, *SENP3‐EIF4A1*, *AC016876.2*, *CD68* and *SENP3*) are in proximity of each other on Chr17.p13. Several putative p53 response elements (RE) have been identified upstream of *CD68* in an *in silico* analysis [31], indicating that p53α may control the expression of these genes via a shared promoter. Three of these overlapping DEGs, *EIF4A1*, *SENP3* and snoRNA *SNORA67* (*AC016876*.2) are involved in translation, indicating that changes driving increased migration and invasion in p53α knockdown sublines may be accentuated at the protein level.

Several of the DEGs support the invasive‐migratory phenotype of the MCF‐7 p53α knockdown subline. In MCF‐7‐shp53α, increased expression of *LRG1* and *HYOU1* (Fig. 5A, Table S1; expression trends confirmed by RT‐qPCR: Fig. 5I) support increased migration and invasion of the subline (Fig. 4G,H), as both genes have been linked to tumorigenicity and cell migration/invasion [32, 33]. These findings also highlight the tumour suppressing function of p53α. Additionally, increased *HYOU1* [32] and decreased *SESN2* [34, 35] expression in MCF‐7‐shp53α (Fig. 5A, Table S1) support the increased proliferation observed in the subline (Fig. 3J). *SESN2* is a repressor of mTOR signalling and the reduction in its expression increases the activity of the pro‐proliferative signalling pathway [34].

In comparison to only nine DEGs in the MCF‐7‐sh∆40p53 subline (Fig. 5D, Table S2), 246 differentially expressed genes were detected in the ZR75‐1‐sh∆40p53 subline when compared to its’ vector control (Fig. 5E, Table S2), representing around 3.4% (246/7159) of the genes detected and offering a possible explanation for the morphological changes observed in this subline (Fig. 3F), and highlighting cell line specific effects of Δ40p53 on gene expression. GSEA did not yield any significant results (Table S3). DEGs in the ZR75‐1‐sh∆40p53 subline include downregulation of genes involved in cell adhesion and extracellular matrix interaction (e.g. *COL16A1*, *AMOTL1*, *ADAMTSL5*, *SGCD)*, as well as downregulation of genes linked to plasma membrane structure and cytoskeletal organisation (e.g. *BIN3*, *PICK1*, *PHACTR1*, *RHOQ*). This differential expression provides further support for the altered morphology observed in these cells. Only four of the DEGs were common between the two sublines (Fig. 5F, Table S2). The lack of commonality in DEGs highlights that ∆40p53 acts in a cell context‐specific manner. Downregulation of *UBE2QL1* (log_2_(fold change): −1.59; FDR‐adj. *P*‐value: 0.0001; Fig. 5D,I, Table S2), a negative regulator of mTOR pathway activity, offers a possible explanation for increased proliferation (Fig. 3J), as well as the trend towards increased migration and invasion observed in the MCF‐7‐sh∆40p53 subline (Fig. 4G,H). While the same changes in cell behaviour (migration and invasion, Fig. 4I,J) were not observed in the ZR75‐1‐sh∆40p53 subline, deregulation of additional genes involved in proliferation, migration and invasion, such as decreased expression of *LRG1*, *ZMYND8*, *GNA13*, *DHX29* and increased expression of *EMILIN2*, and *RECK* may be counteracting the reduced levels of *UBE2QL1* (Fig. 5E, Table S2) by inhibiting proliferation, migration and invasion [36, 37, 38, 39, 40, 41].

Overall, several of the differentially expressed genes in ∆40p53 knockdown sublines support the hypothesis that ∆40p53 acts as a tumour suppressor. Potential oncogenes upregulated in the ∆40p53 knockdown sublines include *EDN1* and *CCDC28B* in the MCF‐7 subline; and *LIFR*, *HOXA11‐AS*, *NACC1*, *FLT4*, *AQP3* and *FAM129A* in ZR75‐1‐sh∆40p53 cells. Similarly, decreased expression of tumour suppressor genes was observed when ∆40p53 was knocked down. Downregulated tumour suppressor genes included *UBE2QL1* in both sublines (expression trend confirmed by RT‐qPCR in MCF‐7 sublines; Fig. 5I); and *SCUBE2*, *PPM1L*, *POLD4*, *SERPINB9*, *WWOX*, *EMSY*, *CCAR2*, *MOB3B*, *PCAT19*, *QSOX1*, *SLX1A*, *PFDN5*, *AMOTL1* and *HERC1* in the ZR75‐1‐sh∆40p53 subline (Fig. 5D,E, Table S2).

Overexpression of ∆40p53 in MCF‐7 cells resulted in the differential expression of 25 genes compared to MCF‐7‐LeGO cells (Fig. 5G, Table S4). Several of the DEGs, such as decreased expression of *KLK7* and *LRG1* (expression trends confirmed by RT‐qPCR; Fig. 5I), and increased expression of *TNKS1BP1*, *PCDH7*, and *SERPINA5* (expression trends confirmed by RT‐qPCR; Fig. 5I) may be contributing to the reduction in invasive and migratory properties [33, 42, 43, 44, 45] in MCF‐7‐∆40p53 cells (Fig. 4A–F, Table S4). Simultaneously, differential expression of these genes supports the tumour suppressor role of ∆40p53 in MCF‐7 cells. An observation further strengthened by opposing observations in MCF‐7 ∆40p53 knockdown cell lines.

Notably, no common DEGs were found between isoform‐specific knockdown in MCF‐7 sublines at the basal level (Fig. 5H), demonstrating loss of Δ40p53 or p53α alone affected separate gene sets. Together with the fact that Δ40p53‐overexpression uniquely induced the expression of 15 genes (Fig. 5H), these results highlight a p53α‐independent function of Δ40p53, which has also been reported by others [8]. Contrastingly, 19 DEGs were common to both ZR75‐1‐shp53α and ZR75‐1‐shΔ40p53 sublines, indicating some similarity in transactivation capacity between the isoforms, yet even in the ZR75‐1 cells, knockdown of Δ40p53 affected the expression of 227 genes that were not affected by p53α knockdown (data not shown). Further, only two genes (*DICER* and *HLTF*) overlapped between MCF‐7‐shΔ40p53 and MCF‐7‐Δ40p53 (Fig. 5H), indicating that overexpression of Δ40p53 may have different effects on gene expression than physiological levels of the p53 isoform.

## 4 Discussion

Wtp53 is present in most breast cancers, suggesting that the canonical tumour suppressing function is compromised. We have shown previously that ∆40p53 is the mostly highly expressed p53 isoform in breast cancer, besides the full‐length p53α isoform, and that a high Δ40p53 : p53α ratio is associated with worse disease‐free survival in breast cancer patients, unveiling a link between ∆40p53 expression and p53 modulation endogenously [11]. This lead to the hypothesis that Δ40p53 may play a role in breast cancer progression. Gene expression array analysis identified distinct clustering of differentially expressed genes by higher or lower Δ40p53 expression in ER+ cases, but not in ER‐ cases (Fig. 1B). DEGs were mostly associated with immune responses (Table 2), indicating that Δ40p53 may participate in modulating p53α‐mediated immune responses in ER+ tumours. Indeed, another p53 isoform, Δ133p53, has been shown to be associated with immunity, interfering with p53α‐mediated anti‐viral responses, and inducing inflammation and autoimmunity in mouse models [46]. In contrast, downregulated genes in tumours with high Δ40p53 expression were mostly cytoskeletal components such as *ACTN1* and *FBLN1*, supporting Δ40p53’s regulation of cell motility.

When Δ40p53 was overexpressed, p53α protein expression was also enhanced (Fig. 2E), suggesting a role for Δ40p53 in stabilising p53α, potentially by forming a heterotetramer and thus attenuating HDM2‐mediated degradation. This protection phenomenon has been reported in Saos‐2 cells by co‐transfection of ∆40p53, p53α and HDM2 [12].

The canonical function of p53α is to monitor DNA integrity by inducing repair, cell cycle arrest and apoptosis. Loss of p53α or mutation of *TP53* induces proliferation [2]. Consistently, proliferation was accelerated when p53α was knocked down in MCF‐7 cells. Similarly, ∆40p53 knockdown enhanced proliferation, though to a lesser extent, while overexpression of ∆40p53 slightly reduced proliferation in MCF‐7 sublines. These findings propose similar roles for Δ40p53 and p53α in proliferation suppression in MCF‐7 cells (Fig 3I,J). This proliferation suppression by Δ40p53 has been previously demonstrated through transfection of p53‐null cells with ∆40p53 vectors [14]. In ZR75‐1 cells, no differences in proliferation were detected, indicating that these findings are likely cell context‐specific.

p53α regulates cell migration and invasion, mostly indirectly via other cofactors, but the function of Δ40p53 in cell mobility has never been examined [23]. Epithelia‐like breast cancer migration and invasion is generally through a migratory front, which passively drags the following cells [47]. Therefore, scratch wound assays were employed. MCF‐7 cells overexpressing Δ40p53 were less migratory and invasive than MCF‐7‐LeGO cells (Fig. 4A–F), implying a role of Δ40p53 in inhibiting cell mobility. Due to changes in the ability to form confluent monolayers and morphological changes in shRNA‐transduced ZR75‐1 cells (Fig. 3D,F,H), scratch wound assays were considered inaccurate. Transwell assays were therefore performed on shRNA‐transduced MCF‐7 and ZR75‐1 sublines for consistency. Increased migration and invasion were observed in both cell lines transduced with p53α‐shRNA, indicating that p53α is a critical safeguard preventing cell mobility. Δ40p53‐shRNA, on the other hand, mildly impaired ZR75‐1 but not MCF‐7 cell mobility (Fig. 4G–J). The reason could be that there are more extracellular matrix‐associated genes being affected by ∆40p53 in ZR75‐1 cells than in MCF‐7 cells (discussed below).

RNA‐seq analysis at the basal level showed that genes associated with increased proliferation potential and decreased tumour suppression were differentially expressed following p53α‐knockdown (Fig. 5A–C), supporting the functional assays. Taken together, these results suggest that loss of p53α in MCF‐7 cells enhanced tumorigenicity as expected. ∆40p53 was found to differentially regulate genes linked to migration and invasion as well as upregulate tumour suppressor genes and downregulate oncogenes (Fig. 5D–H). Together with the functional data, this suggests that at the basal level, the N terminally truncated isoform ∆40p53 may inhibit migration and invasion, while also controlling proliferation. Δ40p53 has been previously reported to retain tumour suppressor function under stress due to the presence of the second transactivation domain [14]. As such, Δ40p53‐overexpression at the basal level assimilated the function of p53α. Whether this is a result of the stabilising effect ∆40p53 exhibited on p53α is still unclear at this point. Few of the genes differentially expressed in Δ40p53 knockdown and overexpression sublines are known p53 target genes, which is supported by the lack of overlap with p53 knockdown sublines. However, *DICER1*, which was found to be downregulated when ∆40p53 was knocked down (log_2_(fold change) −1.768 and −4.538 in MCF‐7 and ZR75‐1, respectively) as well as when ∆40p53 was overexpressed in MCF‐7 cells (log_2_(fold change) −1.299) contains a p53RE in its promoter [48]. *DICER1* is critical for microRNA (miRNA) maturation, which could post‐transcriptionally regulate mRNA expression [49]. As reviewed by Boominathan *et al*. [48], the p53 family mediates a complicated tumour suppressor miRNA network through *DICER1*, controlling tumour suppressor genes such as *PTEN* as well as metastasis‐associated genes including *ZEB1*. The fact that *DICER1* is downregulated regardless of ∆40p53 knockdown or overexpression implies a ratio of ∆40p53 to p53α could be the key to altering *DICER1* and the associated miRNA network. Additionally, Δ40p53 expression has been linked with enhanced stemness in mouse embryos [18], while as reviewed by Molchadsky *et al* [50], p53 is known to promote differentiation and development. Hence, altering the levels of the isoforms may play a role in regulating cell differentiation. The RNA‐seq results showed that the *FGFR3* (Fibroblast growth factor receptor 3, promoting differentiation) gene was downregulated when Δ40p53 was knocked down in both MCF‐7 and ZR75‐1 (Fig. 5F), highlighting the relationship between Δ40p53 and stemness/differentiation.

Inconsistent results from MCF‐7 and ZR75‐1‐derived sublines may result from differences in the expression of other endogenous regulators. For example, HDM2 expression was reported to be higher in ZR75‐1 cells than MCF‐7 cells [51] and thus may suppress p53α function in this cell line. Δ40p53 is HDM2‐insensitive and, therefore, may have taken over the function of p53α to a greater extent in these cells and provides a possible explanation for greater differential expression observed in ZR75‐1‐shΔ40p53 cells compared to MCF‐7‐shΔ40p53 cells (Fig. 5F). Additionally, DEGs associated with cell adhesion and extracellular matrix organisation in ZR75‐1‐shΔ40p53 cells may explain the altered morphology observed in this subline (Fig. 3F). These DEGs include increased *EMILIN2*, *RECK* and *ADMTSL1*, as well as decreased *LRG1*, *COL16A1*, *QSOX1*, *KIAA0319*, *GNA13*, *AMOTL1*, *ADAMTSL5*, *SGCD* and *PXDN* (Fig. 5E and Table S2). The discrepancy in induction of morphological changes may therefore be cell‐dependent.

This study examined two breast cancer cell lines, and this is not representative of all breast cancer cases and subtypes. In particular, our previous studies have demonstrated that Δ40p53 expression was found to be highest in triple negative breast cancers in which *TP53* was frequently mutated [3, 19]. However, the purpose of the current study was to define the function of Δ40p53 in a wtp53α setting. The role of Δ40p53 in the context of mutant p53α needs to be further investigated through other breast cancer cell lines. The custom shRNAs established as part of this study will be a very useful tool for this.

## 5 Conclusion

In summary, examining the role of Δ40p53 in two ER+ breast cancer cell lines revealed differential effects on cell motility in MCF‐7 cells but not in ZR75‐1 cells. In contrast, p53α, acted to restrain cell motility in both cell lines, suggesting it plays a more dominant regulatory role in this context. Together with the downregulation of putative oncogenes and upregulation of tumour suppressor genes, ∆40p53 stunted proliferation, migration and invasion in MCF‐7 cells, highlighting its cell context‐specific function as a tumour suppressor.

## Conflict of interest

The authors declare no conflict of interest.

### Peer Review

The peer review history for this article is available at https://publons.com/publon/10.1002/1878‐0261.13118.

## Author contribution

XZ, BCM and KAAK conceived and designed the project. XZ, BCM, LSR and KAAK acquired the data. XZ, KG, BCM and KAAK analysed and interpreted the data. XZ, KG, LSR and KAAK wrote the paper. HGC and AWB constructed the Δ40p53‐overexpressing lentiviral vector and generated the MCF‐7‐Δ40p53 subline. AWB and JCB provided insights into data interpretation and feedback to the final draft of the manuscript.

## Supporting information


**Fig. S1.** Morphology of parental MCF‐7 and ZR75‐1 cells.
**Table S1.** Differentially expressed genes in p53α knockdown sublines (MCF‐7 and ZR75‐1).
**Table S2.** Differentially expressed genes in ∆40p53 knockdown sublines (MCF‐7 and ZR75‐1).
**Table S3.** Pathway analysis of differentially expressed genes in ZR75‐1‐shΔ40p53 vs ‐shNT.
**Table S4.** Differentially expressed genes in MCF‐7‐∆40p53 cells compared to MCF‐7‐LeGO cells.

## Data Availability

The data that support the findings of this study are available from the corresponding author Kelly.Kiejda@newcastle.edu.au upon reasonable request.
