# Supplementary material for: Effect of p53 and its N‐terminally truncated isoform, Δ40p53, on breast cancer migration and invasion
Source: Mol Oncol. 2021 Nov 23;16(2):447–65. doi: 10.1002/1878-0261.13118 (PMC8763661; doi:10.1002/1878-0261.13118)
Supplement: Supplementary file 1 — Fig. S1. Morphology of parental MCF‐7 and ZR75‐1 cells. Table S1. Differentially expressed genes in p53α knockdown sublines (MCF‐7 and ZR75‐1). Table S2. Differentially expressed genes in ∆40p53 knockdown sublines (MCF‐7 and ZR75‐1). Table S3. Pathway analysis of differentially expressed genes in ZR75‐1‐shΔ40p53 vs ‐shNT. Table S4. Differentially expressed genes in MCF‐7‐∆40p53 cells compared to MCF‐7‐LeGO cells. [file MOL2-16-447-s001.docx]

**
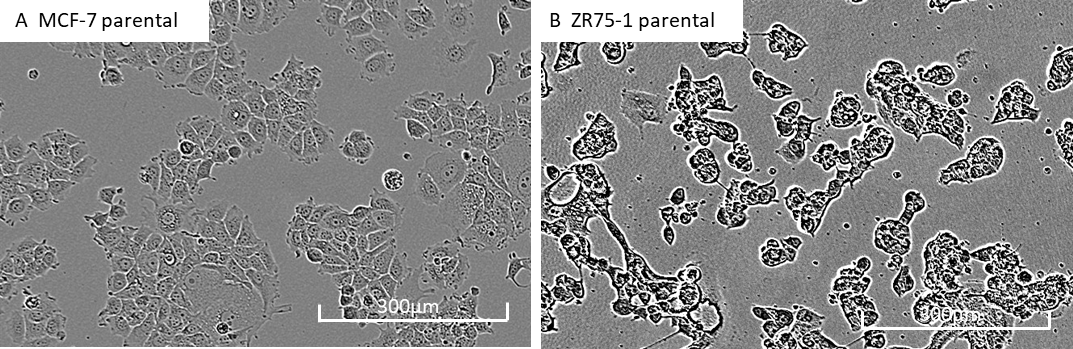
**

**Supplementary Figure S1 Morphology of parental MCF-7 and ZR75-1 cells.**

**Supplementary Table S1: Differentially expressed genes in p53α knockdown sublines (MCF-7 and ZR75-1).**

| p53α knockdown in MCF-7 | | | | | | | |
| --- | --- | --- | --- | --- | --- | --- | --- |
| Gene | Gene Description | baseMean | log_2_(FoldChange) | lfcSE | stat | pvalue | padj |
| LYG2 | Lysozyme G2 | 20 | -1.719 | 0.411 | -4.181 | 2.908E-05 | 8.115E-03 |
| GSE1 | Gse1 Coiled-Coil Protein | 48 | -1.704 | 0.514 | -3.314 | 9.210E-04 | 9.395E-02 |
| AC002463.1 | Novel Transcript | 29 | -1.676 | 0.440 | -3.809 | 1.398E-04 | 2.500E-02 |
| **SENP3-EIF4A1** | **SENP3-EIF4A1 Readthrough** | **460** | **-1.521** | **0.175** | **-8.684** | **3.831E-18** | **2.673E-14** |
| **SENP3** | **SUMO Specific Peptidase 3** | **85** | **-1.431** | **0.301** | **-4.748** | **2.054E-06** | **1.102E-03** |
| SLC1A1 | Solute Carrier Family 1 Member 1 | 222 | -1.427 | 0.188 | -7.610 | 2.749E-14 | 9.588E-11 |
| **EIF4A1** | **Eukaryotic Translation Initiation Factor 4A1** | **581** | **-1.408** | **0.260** | **-5.413** | **6.189E-08** | **5.427E-05** |
| **AC016876.2** | **Small nucleolar RNA SNORA67** | **506** | **-1.325** | **0.308** | **-4.300** | **1.710E-05** | **5.424E-03** |
| AC011379.2 | Novel Transcript | 38 | -1.163 | 0.248 | -4.695 | 2.667E-06 | 1.309E-03 |
| **PRLR** | **Prolactin Receptor** | **2027** | **-1.161** | **0.194** | **-5.977** | **2.277E-09** | **2.647E-06** |
| **CD68** | **CD68 Molecule** | **60** | **-1.081** | **0.323** | **-3.347** | **8.177E-04** | **8.777E-02** |
| ANKRD52 | Ankyrin Repeat Domain 52 | 20 | -1.075 | 0.320 | -3.364 | 7.679E-04 | 8.505E-02 |
| RUNX1T1 | RUNX1 Partner Transcriptional Co-Repressor 1 | 67 | -1.074 | 0.198 | -5.412 | 6.223E-08 | 5.427E-05 |
| ERVK3-1 | Endogenous Retrovirus Group K3 Member 1 | 35 | -1.060 | 0.323 | -3.277 | 1.049E-03 | 9.837E-02 |
| SESN2 | Sestrin 2 | 76 | -1.043 | 0.223 | -4.684 | 2.813E-06 | 1.309E-03 |
| GNE | Glucosamine (UDP-N-Acetyl)-2-Epimerase/N-Acetylmannosamine Kinase | 243 | 1.001 | 0.245 | 4.086 | 4.379E-05 | 1.054E-02 |
| SERPINB9 | Serpin Family B Member 9 | 26 | 1.026 | 0.314 | 3.264 | 1.100E-03 | 9.837E-02 |
| RPS28 | Ribosomal Protein S28 | 94 | 1.206 | 0.286 | 4.214 | 2.503E-05 | 7.278E-03 |
| CEP164 | Centrosomal Protein 164 | 24 | 1.243 | 0.314 | 3.961 | 7.466E-05 | 1.628E-02 |
| PICK1 | Protein Interacting With PRKCA 1 | 135 | 1.276 | 0.259 | 4.934 | 8.071E-07 | 5.631E-04 |
| TBC1D25 | TBC1 Domain Family Member 25 | 32 | 1.409 | 0.389 | 3.625 | 2.887E-04 | 4.378E-02 |
| **LRG1** | **Leucine Rich Alpha-2-Glycoprotein 1** | **76** | **1.515** | **0.376** | **4.027** | **5.649E-05** | **1.271E-02** |
| HYOU1 | Hypoxia Up-Regulated 1 | 83 | 1.551 | 0.439 | 3.533 | 4.115E-04 | 5.417E-02 |
| **PGLYRP4** | **Peptidoglycan Recognition Protein 4** | **13** | **2.176** | **0.559** | **3.890** | **1.001E-04** | **2.054E-02** |
| ZNF681 | Zinc Finger Protein 681 | 21 | 2.366 | 0.470 | 5.029 | 4.929E-07 | 3.821E-04 |
| p53α knockdown in ZR-75-1 | | | | | | | |
| Gene | Gene Description | baseMean | log_2_(FoldChange) | lfcSE | stat | p-value | adj. p-value |
| MATK | Megakaryocyte-Associated Tyrosine Kinase | 78 | -4.327 | 1.315 | -3.291 | 9.978E-04 | 8.974E-02 |
| ACBD6 | Acyl-CoA Binding Domain Containing 6 | 13 | -3.594 | 0.588 | -6.112 | 9.850E-10 | 7.867E-07 |
| ACAP2 | ArfGAP With Coiled-Coil, Ankyrin Repeat And PH Domains 2 | 523 | -1.710 | 0.254 | -6.727 | 1.734E-11 | 1.801E-08 |
| WWOX | WW Domain Containing Oxidoreductase | 24 | -1.638 | 0.436 | -3.755 | 1.734E-04 | 2.466E-02 |
| DAB2 | DAB Adaptor Protein 2 | 26 | -1.574 | 0.310 | -5.073 | 3.921E-07 | 1.804E-04 |
| **SENP3-EIF4A1** | **SENP3-EIF4A1 Readthrough** | **460** | **-1.561** | **0.164** | **-9.544** | **1.371E-21** | **1.424E-17** |
| CRISP2 | Cysteine Rich Secretory Protein 2 | 23 | -1.495 | 0.312 | -4.794 | 1.633E-06 | 6.057E-04 |
| **AC016876.2** | **Small nucleolar RNA SNORA67** | **506** | **-1.434** | **0.284** | **-5.056** | **4.289E-07** | **1.855E-04** |
| **EIF4A1** | **Eukaryotic Translation Initiation Factor 4A1** | **581** | **-1.355** | **0.239** | **-5.673** | **1.407E-08** | **9.130E-06** |
| ABCC11 | ATP Binding Cassette Subfamily C Member 11 | 167 | -1.328 | 0.351 | -3.782 | 1.553E-04 | 2.308E-02 |
| **CD68** | **CD68 Molecule** | **60** | **-1.293** | **0.293** | **-4.413** | **1.017E-05** | **2.779E-03** |
| **PRLR** | **Prolactin Receptor** | **2027** | **-1.289** | **0.189** | **-6.806** | **1.003E-11** | **1.302E-08** |
| **SENP3** | **SUMO Specific Peptidase 3** | **85** | **-1.276** | **0.326** | **-3.911** | **9.185E-05** | **1.572E-02** |
| PNMT | Phenylethanolamine N-Methyltransferase | 25 | -1.204 | 0.342 | -3.517 | 4.359E-04 | 5.202E-02 |
| SLC5A3 | Solute Carrier Family 5 Member 3 | 19 | -1.114 | 0.321 | -3.469 | 5.233E-04 | 5.781E-02 |
| AL022323.5 | Novel Transcript | 130 | -1.081 | 0.236 | -4.586 | 4.509E-06 | 1.419E-03 |
| **LRG1** | **Leucine Rich Alpha-2-Glycoprotein 1** | **76** | **-1.037** | **0.256** | **-4.055** | **5.022E-05** | **9.838E-03** |
| PGAP3 | Post-GPI Attachment to Proteins Phospholipase 3 | 54 | -1.029 | 0.201 | -5.127 | 2.949E-07 | 1.458E-04 |
| PADI6 | Peptidyl Arginine Deiminase 6 | 98 | -1.006 | 0.114 | -8.856 | 8.274E-19 | 4.296E-15 |
| ADAMTSL5 | ADAMTS Like 5 | 59 | 1.006 | 0.179 | 5.636 | 1.738E-08 | 1.061E-05 |
| PAPOLA | Poly(A) Polymerase Alpha | 37 | 1.057 | 0.236 | 4.478 | 7.527E-06 | 2.233E-03 |
| MYBBP1A | MYB Binding Protein 1a | 64 | 1.167 | 0.281 | 4.156 | 3.241E-05 | 6.877E-03 |
| AL365181.2 | Novel Transcript | 70 | 1.256 | 0.364 | 3.453 | 5.542E-04 | 5.872E-02 |
| AC118754.1 | Novel Transcript | 50 | 1.264 | 0.310 | 4.081 | 4.491E-05 | 9.143E-03 |
| AC021054.1 | NINJ2 Antisense RNA | 8 | 1.280 | 0.383 | 3.346 | 8.200E-04 | 7.957E-02 |
| APCDD1 | APC Down-Regulated 1 | 15 | 1.295 | 0.367 | 3.528 | 4.181E-04 | 5.048E-02 |
| DPYSL2 | Dihydropyrimidinase Like 2 | 42 | 1.311 | 0.305 | 4.305 | 1.670E-05 | 4.276E-03 |
| ATG4B | Autophagy Related 4B Cysteine Peptidase | 41 | 1.317 | 0.187 | 7.060 | 1.661E-12 | 2.464E-09 |
| **PGLYRP4** | **Peptidoglycan Recognition Protein 4** | **13** | **1.425** | **0.328** | **4.343** | **1.405E-05** | **3.742E-03** |
| HMGXB3 | HMG-Box Containing 3 | 15 | 1.438 | 0.359 | 4.002 | 6.268E-05 | 1.183E-02 |
| ANKRD18B | Ankyrin Repeat Domain 18B | 58 | 1.470 | 0.414 | 3.551 | 3.843E-04 | 4.751E-02 |
| PLK5 | Polo Like Kinase 5 | 9 | 1.537 | 0.421 | 3.648 | 2.643E-04 | 3.550E-02 |
| RBM24 | RNA Binding Motif Protein 24 | 95 | 1.617 | 0.354 | 4.573 | 4.812E-06 | 1.470E-03 |
| SCML1 | Scm Polycomb Group Protein Like 1 | 26 | 1.652 | 0.398 | 4.156 | 3.245E-05 | 6.877E-03 |
| RTTN | Rotatin | 9 | 1.841 | 0.436 | 4.219 | 2.449E-05 | 5.778E-03 |
| LINC02575 | Long Intergenic Non-Protein Coding RNA 2575 | 22 | 1.852 | 0.532 | 3.480 | 5.023E-04 | 5.669E-02 |
| TTLL1 | Tubulin Tyrosine Ligase Like 1 | 14 | 1.973 | 0.531 | 3.714 | 2.044E-04 | 2.868E-02 |
| AP001065.1 | Z6 small nucleolar RNA | 28 | 2.208 | 0.575 | 3.837 | 1.244E-04 | 1.986E-02 |
| PLXDC1 | Plexin Domain Containing 1 | 8 | 2.231 | 0.639 | 3.494 | 4.765E-04 | 5.498E-02 |
| KLRD1 | Killer Cell Lectin Like Receptor D | 4 | 2.249 | 0.681 | 3.300 | 9.657E-04 | 8.874E-02 |
| PLEKHG4B | Pleckstrin Homology And RhoGEF Domain Containing G4B | 11 | 2.273 | 0.361 | 6.289 | 3.191E-10 | 2.761E-07 |
| RECK | Reversion Inducing Cysteine Rich Protein With Kazal Motifs | 9 | 2.383 | 0.665 | 3.583 | 3.403E-04 | 4.338E-02 |
| AC096763.1 | Novel Transcript | 11 | 2.990 | 0.443 | 6.747 | 1.505E-11 | 1.736E-08 |
| RASL10B | RAS Like Family 10 Member B | 4 | 3.305 | 0.697 | 4.740 | 2.137E-06 | 7.573E-04 |
| POLD4 | DNA Polymerase Delta 4, Accessory Subunit | 10 | 3.323 | 0.406 | 8.188 | 2.660E-16 | 9.207E-13 |
| AP003419.2 | Novel Transcript | 9 | 3.415 | 0.445 | 7.673 | 1.677E-14 | 3.482E-11 |
| AC015631.1 | Novel Transcript | 5 | 3.766 | 0.816 | 4.614 | 3.959E-06 | 1.285E-03 |
| BMF | Bcl2 Modifying Factor | 6 | 4.386 | 1.319 | 3.325 | 8.855E-04 | 8.359E-02 |
| AC044784.1 | Novel Transcript | 3 | 6.238 | 1.573 | 3.966 | 7.311E-05 | 1.309E-02 |

Differentially expressed genes compared to the sublines relative control (i.e. MCF-7-shp53α vs MCF-7-shNT). Genes highlighted in blue are down-regulated and genes highlighted in red are up-regulated compared to the control subline. Genes highlighted in bold are differentially expressed in both MCF-7-shp53α and ZR-75-1-shp53α. adj. – adjusted; baseMean – mean normalised counts in the reference group (MCF-7-shNT or ZR-75-1-shNT); lfcSE – log_2_(Fold Change) standard error; stat – Wald statistic.

**Supplementary Table S2: Differentially expressed genes in ∆40p53 knockdown sublines (MCF-7 and ZR75-1).**

| ∆40p53 knockdown in MCF-7 | | | | | | | |
| --- | --- | --- | --- | --- | --- | --- | --- |
| Gene | Gene Descriptiom | baseMean | log2FoldChange | lfcSE | stat | pvalue | padj |
| PECR | Peroxisomal Trans-2-Enoyl-CoA Reductase | 14 | -5.112 | 1.257 | -4.068 | 4.74E-05 | 3.986E-02 |
| **DICER1** | **Dicer 1, Ribonuclease III** | **377** | **-1.768** | **0.354** | **-5.001** | **5.71E-07** | **1.057E-03** |
| **UBE2QL1** | **Ubiquitin Conjugating Enzyme E2 Q Family Like 1** | **120** | **-1.585** | **0.284** | **-5.580** | **2.410E-08** | **1.114E-04** |
| **HLTF** | **Helicase Like Transcription Factor** | **28** | **-1.353** | **0.338** | **-4.008** | **6.128E-05** | **4.360E-02** |
| CTNND2 | Catenin Delta 2 | 177 | -1.075 | 0.153 | -7.030 | 2.061E-12 | 1.906E-08 |
| **FGFR3** | **Fibroblast Growth Factor Receptor 3** | **69** | **-1.023** | **0.270** | **-3.792** | **1.492E-04** | **8.316E-02** |
| CCDC28B | Coiled-Coil Domain Containing 28B | 61 | 1.344 | 0.355 | 3.786 | 1.529E-04 | 8.316E-02 |
| EDN1 | Endothelin 1 | 86 | 1.760 | 0.361 | 4.882 | 1.049E-06 | 1.617E-03 |
| PMS1 | PMS1 Homolog 1, Mismatch Repair System Component | 6 | 4.019 | 1.077 | 3.733 | 1.890E-04 | 9.710E-02 |
| ∆40p53 knockdown in ZR-75-1 | | | | | | | |
| Gene | Gene Descriptiom | baseMean | log2FoldChange | lfcSE | stat | pvalue | padj |
| AC068580.3 | Novel Transcript | 26 | -6.930 | 1.060 | -6.536 | 6.305E-11 | 1.140E-08 |
| AC037459.4 | Novel Transcript | 42 | -6.568 | 1.315 | -4.995 | 5.871E-07 | 4.301E-05 |
| PFKFB2 | 6-Phosphofructo-2-Kinase/Fructose-2,6-Biphosphatase 2 | 30 | -6.498 | 1.237 | -5.254 | 1.488E-07 | 1.250E-05 |
| SLC26A3 | Solute Carrier Family 26 Member 3 | 24 | -6.485 | 1.282 | -5.057 | 4.251E-07 | 3.262E-05 |
| SCUBE2 | Signal Peptide, CUB Domain And EGF Like Domain Containing 2 | 10 | -6.461 | 1.223 | -5.283 | 1.273E-07 | 1.086E-05 |
| **UBE2QL1** | **Ubiquitin Conjugating Enzyme E2 Q Family Like 1** | **120** | **-6.418** | **1.059** | **-6.063** | **1.336E-09** | **1.828E-07** |
| PPM1L | Protein Phosphatase, Mg2+/Mn2+ Dependent 1L | 312 | -6.248 | 1.105 | -5.656 | 1.545E-08 | 1.570E-06 |
| TPSD1 | Tryptase Delta 1 | 28 | -6.118 | 1.267 | -4.830 | 1.362E-06 | 9.137E-05 |
| AC012456.2 | Novel Transcript | 33 | -6.105 | 1.033 | -5.912 | 3.389E-09 | 4.156E-07 |
| AC106875.1 | Uncharacterized LOC100506405 | 17 | -6.024 | 1.223 | -4.926 | 8.377E-07 | 5.841E-05 |
| DMAC2 | Distal Membrane Arm Assembly Complex 2 | 155 | -5.934 | 0.335 | -17.700 | 4.196E-70 | 2.238E-66 |
| POLD4 | DNA Polymerase Delta 4, Accessory Subunit | 10 | -5.905 | 1.244 | -4.748 | 2.053E-06 | 1.327E-04 |
| BIN3 | Bridging Integrator 3 | 12 | -5.660 | 1.290 | -4.389 | 1.138E-05 | 6.048E-04 |
| TMEM72-AS1 | TMEM72 Antisense RNA 1 | 13 | -5.488 | 1.290 | -4.255 | 2.087E-05 | 9.766E-04 |
| B3GALNT1 | Beta-1,3-N-Acetylgalactosaminyltransferase 1 | 71 | -5.483 | 1.250 | -4.387 | 1.147E-05 | 6.056E-04 |
| AC068580.4 | Peptidase A1 Domain-Containing Protein | 14 | -5.480 | 0.912 | -6.009 | 1.871E-09 | 2.464E-07 |
| PICK1 | Protein Interacting With PRKCA 1 | 135 | -5.388 | 0.372 | -14.488 | 1.436E-47 | 2.553E-44 |
| C8orf58 | Chromosome 8 Open Reading Frame 58 | 16 | -5.327 | 0.911 | -5.848 | 4.967E-09 | 5.636E-07 |
| SERPINB9 | Serpin Family B Member 9 | 26 | -5.298 | 1.229 | -4.311 | 1.624E-05 | 8.208E-04 |
| LPIN1 | Lipin 1 | 282 | -5.282 | 0.480 | -10.996 | 4.016E-28 | 3.570E-25 |
| WWOX | WW Domain Containing Oxidoreductase | 24 | -5.264 | 0.820 | -6.419 | 1.370E-10 | 2.356E-08 |
| UMPS | Uridine Monophosphate Synthetase | 15 | -5.259 | 1.384 | -3.801 | 1.443E-04 | 5.363E-03 |
| EMSY | EMSY Transcriptional Repressor, BRCA2 Interacting | 26 | -5.115 | 0.752 | -6.800 | 1.048E-11 | 2.237E-09 |
| AC125421.2 | Novel Transcript | 23 | -5.108 | 0.861 | -5.936 | 2.914E-09 | 3.615E-07 |
| SPINK4 | Serine Peptidase Inhibitor Kazal Type 4 | 11 | -5.093 | 1.275 | -3.996 | 6.441E-05 | 2.663E-03 |
| CCAR2 | Cell Cycle And Apoptosis Regulator 2 | 65 | -5.020 | 0.600 | -8.365 | 6.035E-17 | 2.384E-14 |
| ZNF41 | Zinc Finger Protein 41 | 166 | -4.922 | 1.122 | -4.389 | 1.140E-05 | 6.048E-04 |
| TMEFF2 | Transmembrane Protein With EGF Like And Two Follistatin Like Domains 2 | 19 | -4.896 | 1.338 | -3.660 | 2.518E-04 | 8.666E-03 |
| AC120498.2 | Novel Transcript, Intronic To TPSD1 | 16 | -4.848 | 1.315 | -3.686 | 2.278E-04 | 7.968E-03 |
| AC012456.1 | Novel Transcript | 24 | -4.731 | 0.804 | -5.887 | 3.943E-09 | 4.674E-07 |
| ELOVL2-AS1 | ELOVL2 Antisense RNA 1 | 20 | -4.720 | 1.279 | -3.690 | 2.246E-04 | 7.880E-03 |
| CTSD | Cathepsin D | 28 | -4.645 | 0.532 | -8.736 | 2.407E-18 | 1.156E-15 |
| **DICER1** | **Dicer 1, Ribonuclease III** | **377** | **-4.538** | **0.813** | **-5.580** | **2.403E-08** | **2.352E-06** |
| CUBN | Cubilin | 15 | -4.478 | 1.276 | -3.508 | 4.511E-04 | 1.463E-02 |
| C2CD4C | C2 Calcium Dependent Domain Containing 4C | 10 | -4.471 | 1.280 | -3.493 | 4.782E-04 | 1.527E-02 |
| DMRTA1 | DMRT Like Family A1 | 25 | -4.376 | 0.762 | -5.746 | 9.143E-09 | 9.952E-07 |
| MATK | Megakaryocyte-Associated Tyrosine Kinase | 78 | -4.211 | 1.315 | -3.203 | 1.358E-03 | 3.474E-02 |
| AC037459.2 | Novel Transcript, Antisense To KIAA1967 | 99 | -4.204 | 0.481 | -8.732 | 2.494E-18 | 1.156E-15 |
| PHACTR4 | Phosphatase And Actin Regulator 4 | 16 | -4.160 | 0.756 | -5.502 | 3.753E-08 | 3.512E-06 |
| AC068580.5 | Novel Transcript | 3886 | -4.143 | 0.253 | -16.371 | 3.078E-60 | 8.207E-57 |
| MOB3B | MOB Kinase Activator 3B | 62 | -4.108 | 0.306 | -13.412 | 5.174E-41 | 6.899E-38 |
| DOK7 | Docking Protein 7 | 27 | -3.810 | 0.770 | -4.948 | 7.481E-07 | 5.290E-05 |
| C4orf19 | Chromosome 4 Open Reading Frame 19 | 159 | -3.760 | 0.222 | -16.918 | 3.318E-64 | 1.180E-60 |
| PCAT19 | Prostate Cancer Associated Transcript 19 | 413 | -3.682 | 0.380 | -9.681 | 3.631E-22 | 2.528E-19 |
| LRG1 | Leucine Rich Alpha-2-Glycoprotein 1 | 76 | -3.682 | 0.293 | -12.571 | 3.046E-36 | 3.611E-33 |
| CCL28 | C-C Motif Chemokine Ligand 28 | 83 | -3.661 | 0.204 | -17.939 | 5.801E-72 | 6.188E-68 |
| ZMYND8 | Zinc Finger MYND-Type Containing 8 | 311 | -3.607 | 0.260 | -13.879 | 8.489E-44 | 1.294E-40 |
| PLIN5 | Perilipin 5 | 17 | -3.516 | 0.813 | -4.325 | 1.524E-05 | 7.761E-04 |
| PDXK | Pyridoxal Kinase | 557 | -3.494 | 0.371 | -9.425 | 4.311E-21 | 2.299E-18 |
| SLC10A3 | Solute Carrier Family 10 Member 3 | 59 | -3.447 | 1.079 | -3.195 | 1.401E-03 | 3.515E-02 |
| MUC5B | Mucin 5B, Oligomeric Mucus/Gel-Forming | 28 | -3.424 | 0.488 | -7.014 | 2.320E-12 | 5.265E-10 |
| MYO6 | Myosin VI | 45 | -3.318 | 0.876 | -3.790 | 1.505E-04 | 5.538E-03 |
| ACBD6 | Acyl-CoA Binding Domain Containing 6 | 13 | -3.254 | 0.580 | -5.613 | 1.989E-08 | 1.965E-06 |
| LRRC8C-DT | LRRC8C Divergent Transcript | 26 | -3.185 | 0.540 | -5.896 | 3.722E-09 | 4.461E-07 |
| LRRC8B | Leucine Rich Repeat Containing 8 VRAC Subunit B | 29 | -3.180 | 0.502 | -6.341 | 2.288E-10 | 3.698E-08 |
| RBM24 | RNA Binding Motif Protein 24 | 95 | -3.150 | 0.727 | -4.335 | 1.459E-05 | 7.517E-04 |
| AC097658.3 | Novel Transcript, Antisense To GAB1 | 32 | -3.119 | 0.898 | -3.471 | 5.182E-04 | 1.612E-02 |
| LMCD1 | LIM And Cysteine Rich Domains 1 | 15 | -3.049 | 0.724 | -4.212 | 2.535E-05 | 1.151E-03 |
| AC114488.2 | Novel Transcript, Antisense To COL16A1 | 12 | -2.791 | 0.519 | -5.381 | 7.390E-08 | 6.624E-06 |
| COL16A1 | Collagen Type XVI Alpha 1 Chain | 47 | -2.775 | 0.289 | -9.589 | 8.892E-22 | 5.269E-19 |
| EPN1 | Epsin 1 | 11 | -2.764 | 0.577 | -4.793 | 1.639E-06 | 1.086E-04 |
| CARHSP1 | Calcium Regulated Heat Stable Protein 1 | 88 | -2.687 | 0.398 | -6.743 | 1.548E-11 | 3.058E-09 |
| BAIAP3 | BAI1 Associated Protein 3 | 71 | -2.683 | 0.351 | -7.643 | 2.119E-14 | 6.109E-12 |
| ARHGAP42 | Rho GTPase Activating Protein 42 | 29 | -2.601 | 0.346 | -7.522 | 5.377E-14 | 1.471E-11 |
| TAB2 | TGF-Beta Activated Kinase 1 (MAP3K7) Binding Protein 2 | 22 | -2.583 | 0.391 | -6.600 | 4.108E-11 | 7.554E-09 |
| PNMT | Phenylethanolamine N-Methyltransferase | 25 | -2.576 | 0.384 | -6.706 | 2.007E-11 | 3.882E-09 |
| LMCD1-AS1 | LMCD1 Antisense RNA 1 | 31 | -2.522 | 0.568 | -4.438 | 9.075E-06 | 5.042E-04 |
| AC016168.2 | Novel Transcript | 33 | -2.456 | 0.298 | -8.249 | 1.602E-16 | 6.102E-14 |
| AC022167.2 | Novel Transcript, Antisense To CARHSP1 | 33 | -2.456 | 0.411 | -5.970 | 2.379E-09 | 3.058E-07 |
| QSOX1 | Quiescin Sulfhydryl Oxidase 1 | 79 | -2.424 | 0.424 | -5.722 | 1.051E-08 | 1.121E-06 |
| IRF6 | interferon Regulatory Factor 6 | 64 | -2.393 | 0.393 | -6.084 | 1.175E-09 | 1.650E-07 |
| AC016168.3 | Novel Transcript | 619 | -2.387 | 0.221 | -10.795 | 3.634E-27 | 2.981E-24 |
| VPS26B | VPS26, Retromer Complex Component B | 98 | -2.383 | 0.303 | -7.852 | 4.097E-15 | 1.366E-12 |
| PGLYRP4 | Peptidoglycan Recognition Protein 4 | 13 | -2.339 | 0.506 | -4.619 | 3.848E-06 | 2.359E-04 |
| SLX1A | SLX1 Homolog A, Structure-Specific Endonuclease Subunit | 28 | -2.294 | 0.373 | -6.150 | 7.767E-10 | 1.135E-07 |
| SLX1A-SULT1A3 | SLX1A-SULT1A3 Readthrough (NMD Candidate) | 28 | -2.228 | 0.366 | -6.093 | 1.107E-09 | 1.575E-07 |
| SCAMP5 | Secretory Carrier Membrane Protein 5 | 20 | -2.226 | 0.778 | -2.862 | 4.207E-03 | 8.134E-02 |
| KIAA0319 | Dyslexia-Associated Protein KIAA0319 | 14 | -2.221 | 0.790 | -2.813 | 4.911E-03 | 9.126E-02 |
| PAIP2B | Poly(A) Binding Protein Interacting Protein 2B | 36 | -2.218 | 0.273 | -8.111 | 5.032E-16 | 1.851E-13 |
| PGAP3 | Post-GPI Attachment To Proteins Phospholipase 3 | 54 | -2.202 | 0.223 | -9.884 | 4.903E-23 | 3.735E-20 |
| BASP1 | Brain Abundant Membrane Attached Signal Protein 1 | 560 | -2.195 | 0.756 | -2.903 | 3.695E-03 | 7.354E-02 |
| PAPOLA | Poly(A) Polymerase Alpha | 37 | -2.124 | 0.375 | -5.661 | 1.503E-08 | 1.541E-06 |
| DBF4 | DBF4 Zinc Finger | 14 | -2.040 | 0.427 | -4.772 | 1.824E-06 | 1.193E-04 |
| ENTPD1-AS1 | ENTPD1 Antisense RNA 1 | 12 | -1.946 | 0.364 | -5.347 | 8.924E-08 | 7.867E-06 |
| AC064805.2 | Novel Transcript, Antisense To RAB37 | 17 | -1.939 | 0.298 | -6.513 | 7.343E-11 | 1.305E-08 |
| PFDN5 | Prefoldin Subunit 5 | 11669 | -1.875 | 0.128 | -14.658 | 1.205E-48 | 2.570E-45 |
| AL031674.1 | Novel Transcript | 11 | -1.858 | 0.433 | -4.289 | 1.793E-05 | 8.704E-04 |
| PHACTR1 | Phosphatase And Actin Regulator 1 | 11 | -1.854 | 0.474 | -3.907 | 9.328E-05 | 3.685E-03 |
| ESPL1 | Extra Spindle Pole Bodies Like 1, Separase | 13164 | -1.837 | 0.238 | -7.727 | 1.098E-14 | 3.405E-12 |
| MRPL55 | Mitochondrial Ribosomal Protein L55 | 67 | -1.832 | 0.228 | -8.029 | 9.852E-16 | 3.390E-13 |
| CCNO | Cyclin O | 98 | -1.798 | 0.187 | -9.634 | 5.720E-22 | 3.589E-19 |
| ELOVL2 | ELOVL Fatty Acid Elongase 2 | 619 | -1.770 | 0.229 | -7.715 | 1.206E-14 | 3.573E-12 |
| NNT | Nicotinamide Nucleotide Transhydrogenase | 215 | -1.692 | 0.210 | -8.062 | 7.506E-16 | 2.669E-13 |
| GNA13 | G Protein Subunit Alpha 13 | 25 | -1.685 | 0.554 | -3.038 | 2.378E-03 | 5.375E-02 |
| DHX29 | DExH-Box Helicase 29 | 12 | -1.670 | 0.541 | -3.085 | 2.036E-03 | 4.711E-02 |
| AC073611.2 | n/a | 4652 | -1.667 | 0.145 | -11.520 | 1.043E-30 | 1.011E-27 |
| RAB31 | RAB31, Member RAS Oncogene Family | 1021 | -1.581 | 0.163 | -9.677 | 3.792E-22 | 2.528E-19 |
| CDIPT | CDP-Diacylglycerol--Inositol 3-Phosphatidyltransferase | 95 | -1.560 | 0.476 | -3.274 | 1.061E-03 | 2.893E-02 |
| AMOTL1 | Angiomotin Like 1 | 38 | -1.559 | 0.216 | -7.200 | 6.023E-13 | 1.397E-10 |
| UGT3A2 | UDP Glycosyltransferase Family 3 Member A2 | 91 | -1.558 | 0.253 | -6.163 | 7.137E-10 | 1.057E-07 |
| SYNE1 | Spectrin Repeat Containing Nuclear Envelope Protein 1 | 78 | -1.554 | 0.232 | -6.703 | 2.038E-11 | 3.882E-09 |
| AC011462.5 | Uncharacterized LOC105372401 | 104 | -1.515 | 0.432 | -3.506 | 4.556E-04 | 1.473E-02 |
| AC026704.1 | Novel Transcript | 13 | -1.508 | 0.351 | -4.300 | 1.706E-05 | 8.427E-04 |
| KRT74 | Keratin 74 | 111 | -1.506 | 0.395 | -3.811 | 1.382E-04 | 5.174E-03 |
| SLCO4C1 | Solute Carrier Organic Anion Transporter Family Member 4C1 | 13 | -1.492 | 0.518 | -2.879 | 3.990E-03 | 7.838E-02 |
| PRKRA-AS1 | Cholesterol Induced Regulator Of Metabolism RNA | 20 | -1.474 | 0.282 | -5.228 | 1.715E-07 | 1.407E-05 |
| CBX6 | Chromobox 6 | 22 | -1.473 | 0.342 | -4.307 | 1.656E-05 | 8.272E-04 |
| WDR90 | WD Repeat Domain 90 | 65 | -1.470 | 0.246 | -5.977 | 2.273E-09 | 2.956E-07 |
| GLYATL3 | Glycine-N-Acyltransferase Like 3 | 62 | -1.464 | 0.500 | -2.926 | 3.429E-03 | 6.993E-02 |
| AC092384.3 | Novel Transcript | 17 | -1.443 | 0.488 | -2.955 | 3.122E-03 | 6.548E-02 |
| FGF12-AS2 | FGF12 Antisense RNA 2 | 40 | -1.442 | 0.414 | -3.480 | 5.007E-04 | 1.580E-02 |
| AC092944.1 | Novel Transcript | 22 | -1.428 | 0.464 | -3.080 | 2.071E-03 | 4.770E-02 |
| KCNB1 | Potassium Voltage-Gated Channel Subfamily B Member 1 | 1684 | -1.424 | 0.240 | -5.937 | 2.904E-09 | 3.615E-07 |
| DPEP3 | Dipeptidase 3 | 41 | -1.415 | 0.203 | -6.960 | 3.407E-12 | 7.571E-10 |
| NBR1 | NBR1 Autophagy Cargo Receptor | 350 | -1.414 | 0.117 | -12.049 | 1.969E-33 | 2.101E-30 |
| CDKL3 | Cyclin Dependent Kinase Like 3 | 41 | -1.395 | 0.194 | -7.201 | 5.986E-13 | 1.397E-10 |
| GPR39 | G Protein-Coupled Receptor 39 | 11 | -1.390 | 0.501 | -2.773 | 5.552E-03 | 9.966E-02 |
| PIWIL4 | Piwi Like RNA-Mediated Gene Silencing 4 | 56 | -1.381 | 0.243 | -5.693 | 1.245E-08 | 1.315E-06 |
| LINC00469 | Long Intergenic Non-Protein Coding RNA 469 | 59 | -1.378 | 0.275 | -5.009 | 5.479E-07 | 4.087E-05 |
| AC073611.1 | Novel Transcript, Antisense To PFDN5 And C12orf10 | 1991 | -1.372 | 0.249 | -5.511 | 3.567E-08 | 3.368E-06 |
| ZNF674 | Zinc Finger Protein 674 | 15 | -1.370 | 0.350 | -3.913 | 9.119E-05 | 3.616E-03 |
| GAREM1 | GRB2 Associated Regulator Of MAPK1 Subtype 1 | 79 | -1.337 | 0.247 | -5.414 | 6.168E-08 | 5.623E-06 |
| NECAB2 | N-Terminal EF-Hand Calcium Binding Protein 2 | 51 | -1.329 | 0.333 | -3.993 | 6.516E-05 | 2.673E-03 |
| AC020915.1 | Novel Transcript, Sense Intronic To ZNF544 | 11 | -1.327 | 0.468 | -2.837 | 4.558E-03 | 8.574E-02 |
| ERVK3-1 | Endogenous Retrovirus Group K3 Member 1 | 35 | -1.319 | 0.247 | -5.351 | 8.731E-08 | 7.762E-06 |
| ADAMTSL5 | ADAMTS Like 5 | 59 | -1.304 | 0.229 | -5.688 | 1.287E-08 | 1.333E-06 |
| OSBPL9 | Oxysterol Binding Protein Like 9 | 126 | -1.290 | 0.152 | -8.514 | 1.680E-17 | 7.469E-15 |
| AL354861.3 | Novel Transcript | 16 | -1.284 | 0.287 | -4.468 | 7.910E-06 | 4.512E-04 |
| SRRM1 | Serine And Arginine Repetitive Matrix 1 | 22 | -1.266 | 0.411 | -3.077 | 2.092E-03 | 4.800E-02 |
| RUNX1T1 | RUNX1 Partner Transcriptional Co-Repressor 1 | 67 | -1.252 | 0.258 | -4.860 | 1.172E-06 | 8.012E-05 |
| RIC8A | RIC8 Guanine Nucleotide Exchange Factor A | 796 | -1.240 | 0.148 | -8.380 | 5.282E-17 | 2.254E-14 |
| INSM1 | INSM Transcriptional Repressor 1 | 77 | -1.231 | 0.361 | -3.410 | 6.506E-04 | 1.955E-02 |
| HIST1H2BC | H2B Clustered Histone 4 | 24 | -1.228 | 0.384 | -3.197 | 1.390E-03 | 3.513E-02 |
| CCDC97 | Coiled-Coil Domain Containing 97 | 26 | -1.216 | 0.262 | -4.641 | 3.460E-06 | 2.146E-04 |
| CDKN2B | Cyclin Dependent Kinase Inhibitor 2B | 81 | -1.215 | 0.301 | -4.037 | 5.418E-05 | 2.312E-03 |
| AC010642.2 | Novel Transcript, Antisense To ERVK3-1 | 27 | -1.212 | 0.280 | -4.325 | 1.528E-05 | 7.761E-04 |
| BX284668.5 | Novel Transcript | 207 | -1.203 | 0.239 | -5.041 | 4.634E-07 | 3.506E-05 |
| SOX13 | SRY-Box Transcription Factor 13 | 38 | -1.185 | 0.218 | -5.447 | 5.132E-08 | 4.761E-06 |
| AL356750.1 | TEC | 178 | -1.174 | 0.370 | -3.171 | 1.517E-03 | 3.704E-02 |
| RETREG1 | Reticulophagy Regulator 1 | 173 | -1.165 | 0.161 | -7.214 | 5.424E-13 | 1.315E-10 |
| DTWD2 | DTW Domain Containing 2 | 30 | -1.161 | 0.313 | -3.714 | 2.043E-04 | 7.239E-03 |
| NPR3 | Natriuretic Peptide Receptor 3 | 125 | -1.161 | 0.202 | -5.759 | 8.475E-09 | 9.320E-07 |
| FOXRED1 | FAD Dependent Oxidoreductase Domain Containing 1 | 90 | -1.155 | 0.203 | -5.691 | 1.262E-08 | 1.320E-06 |
| **FGFR3** | **Fibroblast Growth Factor Receptor 3** | **69** | **-1.125** | **0.212** | **-5.307** | **1.115E-07** | **9.669E-06** |
| RWDD2A | RWD Domain Containing 2A | 13 | -1.118 | 0.330 | -3.386 | 7.084E-04 | 2.093E-02 |
| AC012313.6 | Novel Transcript, Antisense To ZNF497 | 13 | -1.118 | 0.369 | -3.027 | 2.472E-03 | 5.551E-02 |
| PRSS50 | Serine Protease 50 | 33 | -1.112 | 0.319 | -3.485 | 4.921E-04 | 1.567E-02 |
| HAUS5 | HAUS Augmin Like Complex Subunit 5 | 712 | -1.109 | 0.292 | -3.797 | 1.463E-04 | 5.420E-03 |
| SGCD | Sarcoglycan Delta | 55 | -1.106 | 0.243 | -4.561 | 5.088E-06 | 3.032E-04 |
| NEU3 | Neuraminidase 3 | 14 | -1.099 | 0.365 | -3.012 | 2.596E-03 | 5.730E-02 |
| RHOQ | Ras Homolog Family Member Q | 21 | -1.099 | 0.344 | -3.195 | 1.401E-03 | 3.515E-02 |
| TMEM222 | Transmembrane Protein 222 | 63 | -1.090 | 0.229 | -4.763 | 1.911E-06 | 1.243E-04 |
| HERC1 | HECT And RLD Domain Containing E3 Ubiquitin Protein Ligase Family Member 1 | 1013 | -1.078 | 0.142 | -7.569 | 3.764E-14 | 1.057E-11 |
| PRPSAP1 | Phosphoribosyl Pyrophosphate Synthetase Associated Protein 1 | 60 | -1.068 | 0.315 | -3.389 | 7.009E-04 | 2.083E-02 |
| RALGAPA2 | Ral GTPase Activating Protein Catalytic Subunit Alpha 2 | 27 | -1.067 | 0.366 | -2.916 | 3.544E-03 | 7.107E-02 |
| AHRR | Aryl-Hydrocarbon Receptor Repressor | 77 | -1.056 | 0.347 | -3.039 | 2.374E-03 | 5.375E-02 |
| ECH1 | Enoyl-CoA Hydratase 1 | 80 | -1.055 | 0.203 | -5.208 | 1.914E-07 | 1.547E-05 |
| LINC00426 | Long Intergenic Non-Protein Coding RNA 426 | 1798 | -1.055 | 0.264 | -4.001 | 6.299E-05 | 2.614E-03 |
| NEDD9 | Neural Precursor Cell Expressed, Developmentally Down-Regulated 9 | 115 | -1.045 | 0.286 | -3.661 | 2.514E-04 | 8.666E-03 |
| PXDN | Peroxidasin | 535 | -1.045 | 0.178 | -5.875 | 4.233E-09 | 4.962E-07 |
| AL050343.1 | SNORA26L6 | 14 | -1.041 | 0.369 | -2.825 | 4.731E-03 | 8.837E-02 |
| AC104534.1 | Novel Transcript, Antisense To ECH1 | 92 | -1.029 | 0.304 | -3.381 | 7.217E-04 | 2.121E-02 |
| ZNF8 | Zinc Finger Protein 8 | 54 | -1.024 | 0.259 | -3.954 | 7.675E-05 | 3.101E-03 |
| SPCS2 | Signal Peptidase Complex Subunit 2 | 73 | -1.023 | 0.225 | -4.545 | 5.506E-06 | 3.263E-04 |
| CMBL | Carboxymethylenebutenolidase Homolog | 972 | 1.001 | 0.207 | 4.845 | 1.263E-06 | 8.529E-05 |
| AC004477.1 | Novel Transcript, Antisense To NFE2L1 | 12 | 1.010 | 0.342 | 2.950 | 3.177E-03 | 6.606E-02 |
| CES4A | Carboxylesterase 4A | 24 | 1.012 | 0.251 | 4.025 | 5.691E-05 | 2.399E-03 |
| IRX4 | Iroquois Homeobox 4 | 88 | 1.028 | 0.303 | 3.395 | 6.854E-04 | 2.042E-02 |
| AC012640.2 | Novel Transcript, Antisense To MARCH6 | 48 | 1.029 | 0.325 | 3.171 | 1.518E-03 | 3.704E-02 |
| ANKRD34C | Ankyrin Repeat Domain 34C | 30 | 1.035 | 0.222 | 4.672 | 2.983E-06 | 1.872E-04 |
| LIFR | LIF Receptor Subunit Alpha | 147 | 1.040 | 0.242 | 4.290 | 1.788E-05 | 8.704E-04 |
| UBAC1 | UBA Domain Containing 1 | 359 | 1.052 | 0.260 | 4.051 | 5.095E-05 | 2.192E-03 |
| LRRC56 | Leucine Rich Repeat Containing 56 | 48 | 1.061 | 0.316 | 3.363 | 7.720E-04 | 2.238E-02 |
| ANKRD24 | Ankyrin Repeat Domain 24 | 15 | 1.066 | 0.365 | 2.918 | 3.528E-03 | 7.087E-02 |
| HOXA11-AS | HOXA11 Antisense RNA | 15 | 1.070 | 0.288 | 3.719 | 2.000E-04 | 7.130E-03 |
| ZDHHC12 | Zinc Finger DHHC-Type Palmitoyltransferase 12 | 23 | 1.095 | 0.317 | 3.455 | 5.498E-04 | 1.682E-02 |
| SLC1A3 | Solute Carrier Family 1 Member 3 | 11 | 1.119 | 0.347 | 3.223 | 1.270E-03 | 3.329E-02 |
| AC079768.3 | Novel Transcript | 65 | 1.120 | 0.293 | 3.818 | 1.346E-04 | 5.068E-03 |
| AC137695.3 | Novel Transcript, Antisense To COPG1 | 48 | 1.121 | 0.242 | 4.624 | 3.773E-06 | 2.326E-04 |
| NACC1 | Nucleus Accumbens Associated 1 | 50 | 1.138 | 0.187 | 6.094 | 1.102E-09 | 1.575E-07 |
| CNTLN | Centlein | 21 | 1.155 | 0.305 | 3.787 | 1.523E-04 | 5.583E-03 |
| DENND1B | DENN Domain Containing 1B | 49 | 1.167 | 0.251 | 4.642 | 3.450E-06 | 2.146E-04 |
| KANK3 | KN Motif And Ankyrin Repeat Domains 3 | 37 | 1.167 | 0.196 | 5.960 | 2.521E-09 | 3.202E-07 |
| KCNF1 | Potassium Voltage-Gated Channel Modifier Subfamily F Member 1 | 8 | 1.185 | 0.332 | 3.571 | 3.561E-04 | 1.183E-02 |
| RPS28 | Ribosomal Protein S28 | 94 | 1.186 | 0.142 | 8.374 | 5.561E-17 | 2.281E-14 |
| AC138430.1 | Novel Transcript, Readthrough Between TM6SF2 And HAPLN4 | 11 | 1.188 | 0.398 | 2.986 | 2.825E-03 | 6.149E-02 |
| COPG1 | COPI Coat Complex Subunit Gamma 1 | 161 | 1.192 | 0.204 | 5.843 | 5.137E-09 | 5.768E-07 |
| FLT4 | Fms Related Receptor Tyrosine Kinase 4 | 57 | 1.214 | 0.236 | 5.152 | 2.573E-07 | 2.063E-05 |
| AC009084.2 | n/a | 52 | 1.225 | 0.181 | 6.785 | 1.160E-11 | 2.400E-09 |
| NFE2L1 | Nuclear Factor, Erythroid 2 Like 1 | 14 | 1.226 | 0.282 | 4.354 | 1.339E-05 | 6.932E-04 |
| OR2C3 | Olfactory Receptor Family 2 Subfamily C Member 3 | 7 | 1.229 | 0.397 | 3.091 | 1.993E-03 | 4.631E-02 |
| TLR6 | Toll Like Receptor 6 | 22 | 1.251 | 0.257 | 4.874 | 1.095E-06 | 7.535E-05 |
| APLNR | Apelin Receptor | 15 | 1.289 | 0.320 | 4.029 | 5.610E-05 | 2.375E-03 |
| **HLTF** | **Helicase Like Transcription Factor** | **28** | **1.304** | **0.413** | **3.160** | **1.580E-03** | **3.808E-02** |
| AC093323.3 | n/a | 101 | 1.306 | 0.428 | 3.048 | 2.301E-03 | 5.234E-02 |
| AC022509.4 | Novel Transcript, Antisense To SSPN | 52 | 1.324 | 0.267 | 4.963 | 6.958E-07 | 4.982E-05 |
| AC118754.1 | Novel Transcript | 50 | 1.371 | 0.310 | 4.422 | 9.794E-06 | 5.385E-04 |
| SNAPC2 | Small Nuclear RNA Activating Complex Polypeptide 2 | 8 | 1.392 | 0.502 | 2.772 | 5.572E-03 | 9.966E-02 |
| MYBBP1A | MYB Binding Protein 1a | 64 | 1.396 | 0.279 | 5.007 | 5.525E-07 | 4.092E-05 |
| AQP3 | Aquaporin 3 (Gill Blood Group) | 323 | 1.403 | 0.284 | 4.948 | 7.489E-07 | 5.290E-05 |
| AC010503.2 | Novel Transcript, Antisense To TUBB4A | 5 | 1.418 | 0.487 | 2.911 | 3.597E-03 | 7.186E-02 |
| CYP4F26P | Cytochrome P450 Family 4 Subfamily F Member 26, Pseudogene | 23 | 1.434 | 0.382 | 3.749 | 1.773E-04 | 6.367E-03 |
| NDUFA7 | NADH:Ubiquinone Oxidoreductase Subunit A7 | 34 | 1.445 | 0.192 | 7.505 | 6.123E-14 | 1.593E-11 |
| BACH2 | BTB Domain And CNC Homolog 2 | 14 | 1.454 | 0.338 | 4.306 | 1.659E-05 | 8.272E-04 |
| TLR1 | Toll Like Receptor 1 | 9 | 1.468 | 0.349 | 4.206 | 2.596E-05 | 1.173E-03 |
| HOXA11 | Homeobox A11 | 11 | 1.476 | 0.332 | 4.444 | 8.811E-06 | 4.921E-04 |
| ALG9 | ALG9 Alpha-1,2-Mannosyltransferase | 5 | 1.497 | 0.464 | 3.229 | 1.244E-03 | 3.277E-02 |
| AC010503.1 | Novel Transcript, Antisense To TUBB4A | 11 | 1.498 | 0.366 | 4.087 | 4.377E-05 | 1.898E-03 |
| FAM129A | Family With Sequence Similarity 129 Member A | 14 | 1.511 | 0.363 | 4.163 | 3.135E-05 | 1.399E-03 |
| AP005121.1 | Novel Transcript | 7 | 1.512 | 0.449 | 3.365 | 7.663E-04 | 2.229E-02 |
| HMCES | 5-Hydroxymethylcytosine Binding, ES Cell Specific | 37 | 1.529 | 0.342 | 4.471 | 7.789E-06 | 4.467E-04 |
| OXCT1-AS1 | OXCT1 Antisense RNA 1 | 11 | 1.534 | 0.418 | 3.668 | 2.445E-04 | 8.495E-03 |
| AL365181.2 | Novel Transcript | 70 | 1.551 | 0.361 | 4.290 | 1.783E-05 | 8.704E-04 |
| TUBB4A | Tubulin Beta 4A Class IVa | 41 | 1.574 | 0.260 | 6.049 | 1.461E-09 | 1.973E-07 |
| BCL2L13 | BCL2 Like 13 | 38 | 1.584 | 0.367 | 4.310 | 1.631E-05 | 8.208E-04 |
| ZNF683 | Zinc Finger Protein 683 | 8 | 1.638 | 0.417 | 3.924 | 8.691E-05 | 3.472E-03 |
| EMILIN2 | Elastin Microfibril Interfacer 2 | 10 | 1.639 | 0.505 | 3.247 | 1.165E-03 | 3.098E-02 |
| VSIG2 | V-Set And Immunoglobulin Domain Containing 2 | 7 | 1.646 | 0.462 | 3.567 | 3.615E-04 | 1.194E-02 |
| CASP8AP2 | Caspase 8 Associated Protein 2 | 31 | 1.660 | 0.455 | 3.652 | 2.606E-04 | 8.894E-03 |
| RNF182 | Ring Finger Protein 182 | 7 | 1.684 | 0.549 | 3.066 | 2.171E-03 | 4.949E-02 |
| CD320 | CD320 Molecule | 11 | 1.685 | 0.338 | 4.991 | 6.020E-07 | 4.368E-05 |
| MXRA8 | Matrix Remodeling Associated 8 | 5 | 1.712 | 0.507 | 3.379 | 7.263E-04 | 2.128E-02 |
| NKD1 | NKD Inhibitor Of WNT Signaling Pathway 1 | 4 | 1.763 | 0.509 | 3.464 | 5.317E-04 | 1.644E-02 |
| TNKS1BP1 | Tankyrase 1 Binding Protein 1 | 16 | 1.780 | 0.442 | 4.024 | 5.719E-05 | 2.402E-03 |
| PSG7 | Pregnancy Specific Beta-1-Glycoprotein 7 (Gene/Pseudogene) | 8 | 1.932 | 0.377 | 5.122 | 3.019E-07 | 2.386E-05 |
| AP001065.1 | Z6 small nucleolar RNA | 28 | 1.961 | 0.586 | 3.343 | 8.291E-04 | 2.384E-02 |
| TRG-AS1 | T Cell Receptor Gamma Locus Antisense RNA 1 | 19 | 1.991 | 0.316 | 6.308 | 2.834E-10 | 4.513E-08 |
| GLIPR2 | GLI Pathogenesis Related 2 | 23 | 2.023 | 0.405 | 4.995 | 5.886E-07 | 4.301E-05 |
| HAS2-AS1 | HAS2 Antisense RNA 1 | 3 | 2.132 | 0.624 | 3.418 | 6.299E-04 | 1.904E-02 |
| H1FX-AS1 | H1FX Antisense RNA 1 | 14 | 2.312 | 0.539 | 4.287 | 1.813E-05 | 8.710E-04 |
| DCP1B | Decapping MRNA 1B | 4 | 2.360 | 0.741 | 3.186 | 1.443E-03 | 3.597E-02 |
| TRGV4 | T Cell Receptor Gamma Variable 4 | 6 | 2.407 | 0.436 | 5.517 | 3.440E-08 | 3.276E-06 |
| TARBP1 | TAR (HIV-1) RNA Binding Protein 1 | 3 | 2.468 | 0.834 | 2.960 | 3.077E-03 | 6.505E-02 |
| C1QTNF6 | C1q And TNF Related 6 | 25 | 2.471 | 0.331 | 7.454 | 9.043E-14 | 2.297E-11 |
| RECK | Reversion Inducing Cysteine Rich Protein With Kazal Motifs | 9 | 2.514 | 0.665 | 3.782 | 1.557E-04 | 5.671E-03 |
| ADAMTSL1 | ADAMTS Like 1 | 2 | 2.729 | 0.960 | 2.844 | 4.461E-03 | 8.452E-02 |
| LINC02028 | Long Intergenic Non-Protein Coding RNA 2028 | 3 | 2.763 | 0.931 | 2.966 | 3.015E-03 | 6.471E-02 |
| SSTR3 | Somatostatin Receptor 3 | 2 | 2.855 | 0.995 | 2.870 | 4.102E-03 | 8.014E-02 |
| FSD1 | Fibronectin Type III And SPRY Domain Containing 1 | 14 | 3.273 | 1.106 | 2.960 | 3.080E-03 | 6.505E-02 |
| AKR1C2 | Aldo-Keto Reductase Family 1 Member C2 | 4 | 3.275 | 1.117 | 2.933 | 3.361E-03 | 6.894E-02 |
| AC096763.1 | Novel Transcript | 11 | 3.403 | 0.440 | 7.725 | 1.117E-14 | 3.405E-12 |
| AC015631.1 | Novel Transcript | 5 | 3.749 | 0.818 | 4.586 | 4.524E-06 | 2.726E-04 |
| FBXL7 | F-Box And Leucine Rich Repeat Protein 7 | 4 | 3.892 | 0.944 | 4.124 | 3.723E-05 | 1.641E-03 |
| GDNF-AS1 | GDNF Antisense RNA 1 | 2 | 4.704 | 1.362 | 3.455 | 5.502E-04 | 1.682E-02 |
| BMF | Bcl2 Modifying Factor | 6 | 5.209 | 1.295 | 4.021 | 5.798E-05 | 2.426E-03 |
| ZFR2 | Zinc Finger RNA Binding Protein 2 | 4 | 6.695 | 1.315 | 5.093 | 3.532E-07 | 2.750E-05 |

Differentially expressed genes compared to the sublines relative control (i.e. MCF-7-sh∆40p53 vs MCF-7-shNT). Genes highlighted in blue are down-regulated and genes highlighted in red are up-regulated compared to the control subline. Genes highlighted in bold are differentially expressed in both MCF-7-sh∆40p53 and ZR-75-1-sh∆40p53. adj. – adjusted; baseMean – mean normalised counts in the reference group (MCF-7-shNT or ZR-75-1-shNT); lfcSE – log_2_(Fold Change) standard error; stat – Wald statistic.

**Supplementary Table S3: Pathway analysis of differentially expressed genes in ZR75-1-shΔ40p53 vs -shNT.**

| Term | Overlap | P-value | Adjusted P-value | Odds Ratio | Combined Score | Genes |
| --- | --- | --- | --- | --- | --- | --- |
| negative regulation of developmental growth (GO:0048640) | 3/32 | 0.002 | 0.441 | 12.727 | 77.565 | CDKL3;KIAA0319;FGFR3 |
| positive regulation of lipase activity (GO:0060193) | 2/10 | 0.003 | 0.441 | 30.599 | 178.999 | FGFR3;PLIN5 |
| catecholamine biosynthetic process (GO:0042423) | 2/12 | 0.004 | 0.441 | 24.477 | 134.074 | PNMT;INSM1 |
| positive regulation of calcium ion-dependent exocytosis (GO:0045956) | 2/13 | 0.005 | 0.441 | 22.250 | 118.282 | SCAMP5;KCNB1 |
| positive regulation of regulated secretory pathway (GO:1903307) | 2/17 | 0.008 | 0.441 | 16.314 | 78.004 | SCAMP5;KCNB1 |
| glycolipid metabolic process (GO:0006664) | 3/52 | 0.009 | 0.441 | 7.525 | 35.515 | NEU3;B3GALNT1;PGAP3 |
| sphingolipid metabolic process (GO:0006665) | 4/116 | 0.015 | 0.441 | 4.403 | 18.388 | NEU3;B3GALNT1;PPM1L;ELOVL2 |
| extracellular matrix assembly (GO:0085029) | 2/24 | 0.016 | 0.441 | 11.119 | 45.713 | PXDN;QSOX1 |
| positive regulation of phospholipase activity (GO:0010518) | 2/24 | 0.016 | 0.441 | 11.119 | 45.713 | GNA13;FGFR3 |
| negative regulation of axonogenesis (GO:0050771) | 2/28 | 0.022 | 0.441 | 9.406 | 35.915 | CDKL3;KIAA0319 |
| cellular response to bacterial lipopeptide (GO:0071221) | 2/6 | 0.000 | 0.119 | 124.463 | 1034.086 | TLR1;TLR6 |
| regulation of toll-like receptor 2 signaling pathway (GO:0034135) | 2/11 | 0.001 | 0.216 | 55.303 | 388.361 | TLR1;TLR6 |
| vasculature development (GO:0001944) | 2/17 | 0.002 | 0.308 | 33.172 | 203.445 | FLT4;APLNR |
| negative regulation of protein polymerization (GO:0032272) | 2/27 | 0.005 | 0.308 | 19.893 | 103.673 | TUBB4A;KANK3 |
| MyD88-dependent toll-like receptor signaling pathway (GO:0002755) | 2/29 | 0.006 | 0.308 | 18.418 | 93.400 | TLR1;TLR6 |
| sprouting angiogenesis (GO:0002040) | 2/52 | 0.019 | 0.308 | 9.934 | 39.223 | FLT4;RECK |
| positive regulation of B cell activation (GO:0050871) | 2/53 | 0.020 | 0.308 | 9.739 | 38.103 | HMCES;CD320 |
| positive regulation of anoikis (GO:2000210) | 1/5 | 0.020 | 0.308 | 61.463 | 239.425 | MYBBP1A |
| negative regulation of metallopeptidase activity (GO:1905049) | 1/5 | 0.020 | 0.308 | 61.463 | 239.425 | RECK |
| L-aspartate transmembrane transport (GO:0070778) | 1/5 | 0.020 | 0.308 | 61.463 | 239.425 | SLC1A3 |

Pathways highlighted in blue are attributed to genes that were down-regulated in -shΔ40p53 vs -shNT and pathways highlighted in red are attributed to genes that were up-regulated.

**Supplementary Table S4: Differentially expressed genes in MCF-7-∆40p53 cells compared to MCF-7-LeGO cells.**

| ∆40p53 overexpression in MCF-7 | | | | | | | |
| --- | --- | --- | --- | --- | --- | --- | --- |
| Gene | Gene Descriptiom | baseMean | log2FoldChange | lfcSE | stat | pvalue | padj |
| KLK7 | Kallikrein Related Peptidase 7 | 100 | -5.954 | 1.232 | -4.834 | 1.341E-06 | 8.509E-04 |
| ZNF681 | Zinc Finger Protein 681 | 21 | -1.861 | 0.447 | -4.160 | 3.181E-05 | 9.248E-03 |
| HNRNPL | Heterogeneous Nuclear Ribonucleoprotein L | 29 | -1.526 | 0.346 | -4.405 | 1.060E-05 | 4.577E-03 |
| HLTF | Helicase Like Transcription Factor | 28 | -1.495 | 0.340 | -4.399 | 1.088E-05 | 4.577E-03 |
| LRG1 | Leucine Rich Alpha-2-Glycoprotein 1 | 76 | -1.424 | 0.298 | -4.781 | 1.743E-06 | 1.013E-03 |
| LINC01634 | Long Intergenic Non-Protein Coding RNA 1634 | 56 | -1.338 | 0.316 | -4.227 | 2.364E-05 | 7.496E-03 |
| DICER1 | Dicer 1, Ribonuclease III | 377 | -1.299 | 0.352 | -3.694 | 2.208E-04 | 4.532E-02 |
| MRPL55 | Mitochondrial Ribosomal Protein L55 | 67 | -1.183 | 0.302 | -3.921 | 8.836E-05 | 2.126E-02 |
| CD68 | CD68 Molecule | 60 | 1.124 | 0.237 | 4.746 | 2.074E-06 | 1.113E-03 |
| AC010618.1 | Ribosomal Protein L21 (RPL21) Pseudogene | 314 | 1.210 | 0.210 | 5.766 | 8.116E-09 | 8.090E-06 |
| VAMP1 | Vesicle Associated Membrane Protein 1 | 169 | 1.333 | 0.328 | 4.071 | 4.690E-05 | 1.309E-02 |
| TNKS1BP1 | Tankyrase 1 Binding Protein 1 | 16 | 1.719 | 0.391 | 4.394 | 1.115E-05 | 4.577E-03 |
| EPHA3 | EPH Receptor A3 | 10 | 1.768 | 0.438 | 4.034 | 5.491E-05 | 1.474E-02 |
| BTN3A1 | Butyrophilin Subfamily 3 Member A1 | 37 | 1.897 | 0.500 | 3.797 | 1.466E-04 | 3.196E-02 |
| CRISP2 | Cysteine Rich Secretory Protein 2 | 23 | 1.913 | 0.323 | 5.931 | 3.020E-09 | 3.511E-06 |
| SENP3-EIF4A1 | SENP3-EIF4A1 Readthrough | 460 | 2.018 | 0.140 | 14.462 | 2.107E-47 | 1.470E-43 |
| BIN2 | Bridging Integrator 2 | 26 | 2.108 | 0.455 | 4.634 | 3.581E-06 | 1.784E-03 |
| PCDH7 | Protocadherin 7 | 94 | 2.348 | 0.610 | 3.850 | 1.181E-04 | 2.746E-02 |
| PCGF5 | Polycomb Group Ring Finger 5 | 9.1271021 | 2.779 | 0.763 | 3.642 | 2.710E-04 | 5.401E-02 |
| EIF4A1 | Eukaryotic Translation Initiation Factor 4A1 | 581.13659 | 3.070 | 0.222 | 13.799 | 2.599E-43 | 9.067E-40 |
| AC016876.2 | Novel Transcript | 506.30471 | 3.100 | 0.269 | 11.544 | 7.887E-31 | 1.834E-27 |
| SERPINA5 | Serpin Family A Member 5 | 26.352381 | 3.653 | 0.543 | 6.732 | 1.670E-11 | 2.330E-08 |
| FRMPD1 | FERM And PDZ Domain Containing 1 | 56.824442 | 6.412 | 0.609 | 10.533 | 6.055E-26 | 1.056E-22 |

Differentially expressed genes in MCF-7-∆40p53 vs MCF-7-LeGO. Genes highlighted in blue are down-regulated and genes highlighted in red are up-regulated compared to the control subline. adj. – adjusted; baseMean – mean normalised counts in the reference group (MCF-7-LeGO); lfcSE – log_2_(Fold Change) standard error; stat – Wald statistic.
